# Temperature Shift Between Vineyards Modulates Berry Phenology and Primary Metabolism in a Varietal Collection of Wine Grapevine

**DOI:** 10.3389/fpls.2020.588739

**Published:** 2020-12-17

**Authors:** Kelem Gashu, Noga Sikron Persi, Elyashiv Drori, Eran Harcavi, Nurit Agam, Amnon Bustan, Aaron Fait

**Affiliations:** ^1^French Associates Institute for Agriculture and Biotechnology of Drylands, The Jacob Blaustein Institutes for Desert Research, Ben-Gurion University of the Negev, Beersheba, Israel; ^2^Department of Chemical Engineering, Ariel University, Ariel, Israel; ^3^The Grape and Wine Research Center, Eastern Regional R&D Center, Ariel, Israel; ^4^Ministry of Agriculture and Rural Development, Agricultural Extension Service – Shaham, Beit Dagan, Israel; ^5^Ramat Negev Desert Agro-Research Center, Ramat Negev Works Ltd., Haluza, Israel

**Keywords:** arid viticulture, climate change, organic acids, phenological phase, primary metabolism, sugars, *Vitis vinifera*

## Abstract

Global climate change and the expected increase in temperature are altering the relationship between geography and grapevine (*V. vinifera*) varietal performance, and the implications of which are yet to be fully understood. We investigated berry phenology and biochemistry of 30 cultivars, 20 red and 10 white, across three seasons (2017–2019) in response to a consistent average temperature difference of 1.5°C during the growing season between two experimental sites. The experiments were conducted at Ramat Negev (RN) and Ramon (MR) vineyards, located in the Negev desert, Israel. A significant interaction between vineyard location, season, and variety affected phenology and berry indices. The warmer RN site was generally associated with an advanced phenological course for the white cultivars, which reached harvest up to 2 weeks earlier than at the MR site. The white cultivars also showed stronger correlation between non-consecutive phenological stages than did the red ones. In contrast, harvest time of red cultivars considerably varied according to seasons and sites. Warmer conditions extended fruit developmental phases, causing berry shriveling and cluster collapse in a few cultivars such as Pinot Noir, Ruby Cabernet, and Tempranillo. Analyses of organic acid content suggested differences between red and white cultivars in the content of malate, tartrate, and citrate in response to the temperature difference between sites. However, generally, cultivars at lower temperatures exhibited lower concentrations of pulp organic acids at véraison, but acid degradation until harvest was reduced, compared to the significant pace of acid decline at the warmer site. Sugars showed the greatest differences between sites in both white and red berries at véraison, but differences were seasonal dependent. At harvest, cultivars of both groups exhibited significant variation in hexose/sucrose ratio, and the averages of which varied from 1.6 to 2.9. Hexose/sucrose ratio was significantly higher among the red cultivars at the warmer RN, while this tendency was very slight among white cultivars. White cultivars seem to harbor a considerable degree of resilience due to a combination of earlier and shorter ripening phase, which avoids most of the summer heat. Taken together, our study demonstrates that the extensive genetic capacity of *V. vinifera* bears significant potential and plasticity to withstand the temperature increase associated with climate change.

## Introduction

Most of the world’s viticulture regions are confined to specific geographic niches. Few climatic indices have been employed as metrics to define the boundaries of these regions. However, the recent climate changes considerably threaten the validity of these boundaries, undermining the equilibrium between climate, soil, and variety (Moriondo et al., 2013; Wolkovich et al., 2018; Van Leeuwen et al., 2019). Particularly, the prevalence of recurring years with air temperatures higher than the long-term (30 years) average is disrupting the conservative relationships between geography and viticulture, resulting in remarkable changes in the presently known world wine industry (Fraga et al., 2016; Wolkovich et al., 2018), yet to be fully estimated. In addition, substantial effects on yield and quality along with increases in demand are expected to expand and generate a gradual shift of wine production from traditional regions to newly suitable areas (Hannah et al., 2013; Santillán et al., 2019; Morales-Castilla et al., 2020; Santos et al., 2020).

Recurrent high temperatures tend to diminish wine grape-berry quality traits such as sugars, acids, and phenylpropanoids. Therefore, and in spite of considerable diverse varietal sensitivity to temperature regimes (Gladstones, 1992), warmer regions are predicted to experience the greatest decline in quality and potentially in yield (Moriondo et al., 2013). For example, a recent study, conducted on land suitability for 11 popular cultivars using long-term records, found that a 2°C rise in air temperature might result in 24–56% loss of viticulture area within current wine-growing regions (Morales-Castilla et al., 2020). While efforts have been put to identify the most suitable climate zone for each cultivar (Hall and Jones, 2010; Jones et al., 2012) and to decipher the effect of heat stress on grapes (Jones et al., 2005; Sadras and Moran, 2013), a substantial gap of knowledge exists regarding possible implications of the 2°C rise predicted by climate models on grapevine varietal response, vine and berry phenology, and berry metabolism.

Temperature is known to affect grapevine phenology (Jones et al., 2005; Martínez-Lüscher et al., 2016a; van Leeuwen and Darriet, 2016). For example, accelerated phenological events due to high temperature can shift berry ripening into the warmest part of the season (Webb et al., 2007; Duchêne et al., 2010; Sadras and Moran, 2013; Ruml et al., 2016) and shorten the intervals between phenological phases (Webb et al., 2007; Bock et al., 2011; Tomasi et al., 2011). During fruit ripening, high temperatures reduce the accumulation of anthocyanins (Bergqvist et al., 2001; Pastore et al., 2017; Ramos and Martínez de Toda, 2020) and enhance catabolism of the main organic acids (Lakso and Kliewer, 1975; Sweetman et al., 2014; Rienth et al., 2016) causing a loss of acidity. The effect on berry sugar content remains unclear (Keller, 2010; Reshef et al., 2017). Beyond these general statements, cultivars may differ significantly in many aspects that determine fruit quality, including the timing of bud break, bloom, and véraison, as well as fruit development and ripening processes, when responding to identical sets of environmental conditions (Jones and Davis, 2000; Wolkovich et al., 2018). This broad genetic diversity encompassed by *Vitis vinifera* (Anderson and Aryal, 2013; Moriondo et al., 2013; Real et al., 2015) bears the capacity to provide varieties that can produce high-quality wines also in warm climates (Wolkovich et al., 2018).

Although the grapevine’s phenology responses to climate change have been studied (Duchêne et al., 2010; Parker et al., 2011; De Cortázar-Atauri et al., 2017; Alikadic et al., 2019), a satisfactory understanding of how different wine grape cultivars may respond to a temperature shift is yet beyond reach. Moreover, studies employing artificial warming experiments to examine the effect of temperature on phenology and berry chemical compositions (Cleland et al., 2012; Sadras and Moran, 2013) might fail to provide reliable predictions (Wolkovich et al., 2012).

With the objective to explore the effects of environmental and varietal components modulating berry phenology and metabolism, we tested the effect of a consistent difference of 1.5°C in air temperature on the development and berry indices of 30 wine-grape varieties, 20 red and 10 white, grafted on the same rootstock, grown in vineyard conditions at two distinct arid topo-climatic regions. The advantages of field trials in arid regions are as follows: (i) reliable control of water input, as no rainfall occurs during fruit maturation; (ii) low air humidity and thus low risks of pathogenic hazards (Carroll and Wilcox, 2003; Eastburn et al., 2011); and (iii) a relatively low intra- and inter-seasonal variability. Moreover, significantly large gaps between daily minimum and maximum temperatures (yet within the range for viticulture), abundant clear-sky conditions, and sufficient exposure to sunlight provide suitable conditions for quality fruit development (Bernardo et al., 2018; Ohana-Levi et al., 2020). To our knowledge, this is one of the very few studies of its kind (Tomasi et al., 2011; Ruml et al., 2016).

## Materials and Methods

### Plant Material and Experimental Site

**FIGURE 1:**
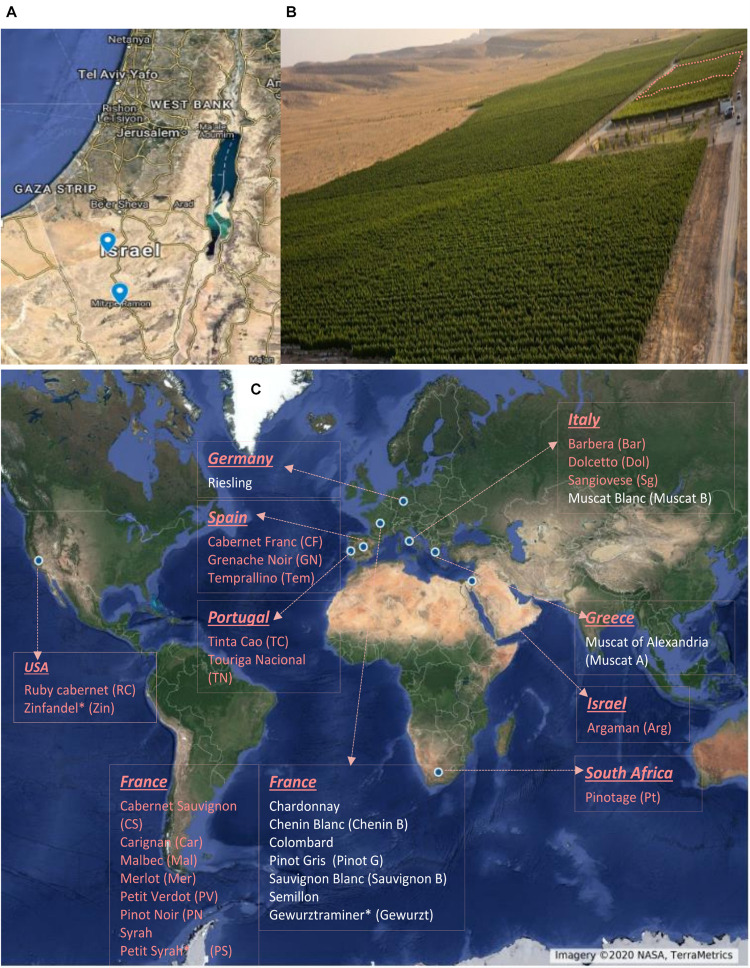
Geographic location of experimental vineyards **(A)**. Aerial view of Ramon vineyard. The experimental area is marked with dashed lines **(B)**. List of cultivars used in the experiment and their origin **(C)**. Names with red and white color denote red and white cultivars, respectively. Cultivar names are composed by abbreviations in the bracket. ^∗^Country of origin not defined.

The experiments were conducted during three consecutive seasons, from 2017 to 2019, in two vineyards in the Negev Highlands in Israel (Figure 1A); Ramon (MR) vineyard (Figure 1B) vineyard (30°38′48.6″N 34°47′24.5″E, 850 m asl) and the Ramat Negev (RN) vineyard at the Desert Agro-Research Center (30°58′43.4″N 34°42′31.6″E, 300 m asl). The two locations are 53-km distant. The average annual precipitation are 105 and 80 mm at MR and RN, respectively, occurring only in the winter (typically November through April), with considerable year-to-year fluctuations. Both vineyards shared the same experimental setup, comprising 30 wine grape (*Vitis vinifera L.*) cultivars (10 white and 20 red; Figure 1C), grafted on 140 RU rootstock. Both vineyards were planted in 2012 in a randomized block design with four replicates of eight–nine vines each (30 cultivars × 2 locations × 4 biological replicates). Each cultivar was represented in each of four independent replicate blocks by at least eight vines (32–36 vines per cultivar, in each vineyard). Phenological assessments and sampling for biochemical analyses were conducted on each cultivar using each of the four replicate blocks (independent biological replicate) in each vineyard, as further detailed below.

The space between rows and between vines was 3 and 1.5 m, respectively. In order to reduce the variation between the vineyards the same rootstock, trellising technique (vertical shoot position, VSP), orientation (north-south), cultural practices, and irrigation systems were used. The soils at both sites are sandy loam. Drip-irrigation systems, mulched with white plastic sheets, as commonly used in the region, supplied about 500 mm each year, from bloom to harvest. Irrigation rate was adjusted weekly according to the current evapotranspiration and crop coefficients. Deficit irrigation was exercised to control vine vigor until véraison (crop coefficient of 0.35) and to impose a moderate water stress during fruit ripening (crop coefficient of 0.25). Yield was adjusted to 10–15 Mg ha^–1^ using appropriate winter pruning, branch, and cluster thinning during the season. The vegetative growth and canopy size were controlled in the VSP design by pruning branches at 2.2 m aboveground. Fertilizer was supplied through the irrigation system, and pest management was carried out according to the common regional recommendations.

### Meteorological Data Measurement

Hourly meteorological data (i.e., incoming solar irradiance, air temperature, relative humidity, and wind speed and direction) were extracted from standard meteorological stations (Meteotech Ltd., Israel) located at the Desert Agro-Research Center, 500 m distant from RN vineyard, and at MR vineyard, during 2017–2019 and 2018–2019 seasons, respectively. The incoming solar irradiance, air temperature, relative humidity, and wind speed and direction were measured continuously at 0.1 Hz using a portable meteorological station (WS501-UMB, Lufft, Fellbach, Germany) set 2 m above the canopy, and 15-min averages were logged by a data logger (CR200, Campbell Scientific, Utah, United States). During the growing season of 2017 (23 May–29 August), this meteorological station was set at MR site, at 2 m above the canopy, and provided 15-min averages of the meteorological conditions within the vineyard.

The Huglin index (HI; Huglin, 1978), a degree-day index used to estimate grapevine thermal exposure during its phenological course (Fraga et al., 2012; Lorenzo et al., 2013; Sánchez et al., 2019), was fitted to the local earlier phenological course and was computed from March to August, instead of the standard April to October grapevine growing season (north hemisphere), using the following equation:

(1) $$\text{HI}=\sum_{\text{March}}^{\text{August}}\left(\frac{\left(\text{MDT}-10\right)+\left(\text{Tmax}-10\right)}{2}k\right),$$

where MDT is the mean daily temperature, Tmax is maximum daily temperature, and *k* is day length coefficient (1.02 to 1.06).

### Phenological Data Collection

The stage of grapevine crop development (E-L scale; Coombe, 1995) was determined weekly on eight–nine vines in each of four replicates of a given cultivar at each location. The timing of four phenological events and the duration of the intervals between them was recorded yearly during 2017–2019 seasons at both vineyards, at the cultivar and replicate levels. These events included the following: (1) date of bud break (BB), at E-L 4 (Coombe, 1995); (2) initial fruit set (FS; E-L 27); (3) véraison (Vér; E-L 35); and (4) harvest (Har; E-L 38). The difference in the durations of similar phenological intervals between MR and RN vineyards was calculated and defined as the phenological shift.

### Berry Sampling and Metabolite Extraction

During each season, berries were sampled at véraison and at harvest for metabolite extraction and berry indices. At véraison, each cultivar’s replicate was sampled when berries reached about 50% color change or softening (estimated weekly in eight tagged representative clusters per replicate), in red or white cultivars, respectively. Toward harvest, berries were sampled at each cultivar’s replicate approaching a specific°Brix level, i.e., 23 ± 1 and 20 ± 1°. Brix, for red and white cultivars, respectively. For each cultivar, samples were collected from four biological replicates at each location, as follows. In each sampling, at least 30 berries per replicate were pooled from five different vines in each block (six berries per vine were sampled from the top, middle, and bottom of the bunch), on the east side of the vine, and immediately snap-frozen in liquid nitrogen. Berries were sectioned while still frozen, skin and pulp carefully separated, and seeds were removed. The pulp was kept at −80°C until further analysis.

### Pulp Organic Acid and Sugar Analysis

Pulp samples were lyophilized and ground under liquid nitrogen using a Retsch-mill (Retsch, Haan, Germany) with prechilled holders and grinding beads. For metabolite extraction, 20 mg of frozen pulp powder was weighed and extracted in a 1-ml pre-cooled methanol/chloroform/water extraction solution (2.5:1:1 *v*/*v*) as described in Hochberg et al. (2013); Degu et al. (2014). Then, 120 μl (véraison) and 100 μl (harvest) of extracts were dried using Concentrator Plus (Eppendorf, Hamburg, Germany) and derivatized exactly as described in Hochberg et al. (2015) with sorbitol as the internal standard. Extracts were injected into the GC-MS for organic acid and sugars analysis. Malate, tartrate, citrate, glucose, fructose, and sucrose were quantified using a calibration curve of standards (Sigma-Aldrich, MO, United States) as described previously by Reshef et al. (2017). The GC-MS conditions were exactly as described previously by Reshef et al. (2019). Analyses were conducted in two consecutive seasons. Since the metabolic response of each cultivar was not always the same between two seasons, a third season (2019) was used as validation and analyzed in bulks of the four replicates × cultivar × location.

Qualitative and Quantitative Data Analysis-Mass Hunter Workstation Software (Agilent Technologies, Santa Clara, CA, United States) was used for integration of peak area and data analysis. Metabolite annotation was performed based on spectral searching supported by the National Institute of Standards and Technology (NIST, Gaithersburg, MA, United States) against RI libraries from the Max Planck Institute for Plant Physiology (Golm, Germany) and finally normalized by the internal standard sorbitol 6C^13^ (Cortecnet Corporation, Mill Valley, CA, United States) and pulp dry weight.

### Statistical Analysis

Statistical analysis was performed using software “R” version 3.6.0 (R Development Core Team, 2017) and JMP^®^, version 13 (SAS institute Inc., Cary, NC, 1989–2007). One-way analysis of variance (ANOVA) was used to assess the genetic variability for each parameter between the seasons within the same location using the built-in *aov* function. The differences between locations for each cultivar were tested using *t.test* and *Wilcox.test* functions according to the distribution of the data. Histograms were created using *hist* function in “ggplot2” package. The wind rose graphs were created using “open air” package (Carslaw and Ropkins, 2012). Clustered heatmaps were created using *Complexheatmap* (Gu et al., 2016). Hierarchical clustering of samples was calculated by Euclidean distances and the Ward.D2 clustering method in functions *get_dist* and *hclust*, built-in “dendextend,” and “factoextra” packages (Galili, 2015). Correlation analysis was performed using *ggscatter* function built in “ggpubr” package. A three-way factorial analysis was performed using JMP^®^, version 13 (SAS institute Inc., Cary, NC, 1989–2007), to assess the interaction effects between cultivar, location, and growing seasons. Principal component analyses were plotted using the software “Metaboanalysit” version 4.0 (Chong et al., 2018).

## Results

### Climatic Conditions in the Vineyards

**FIGURE 2:**
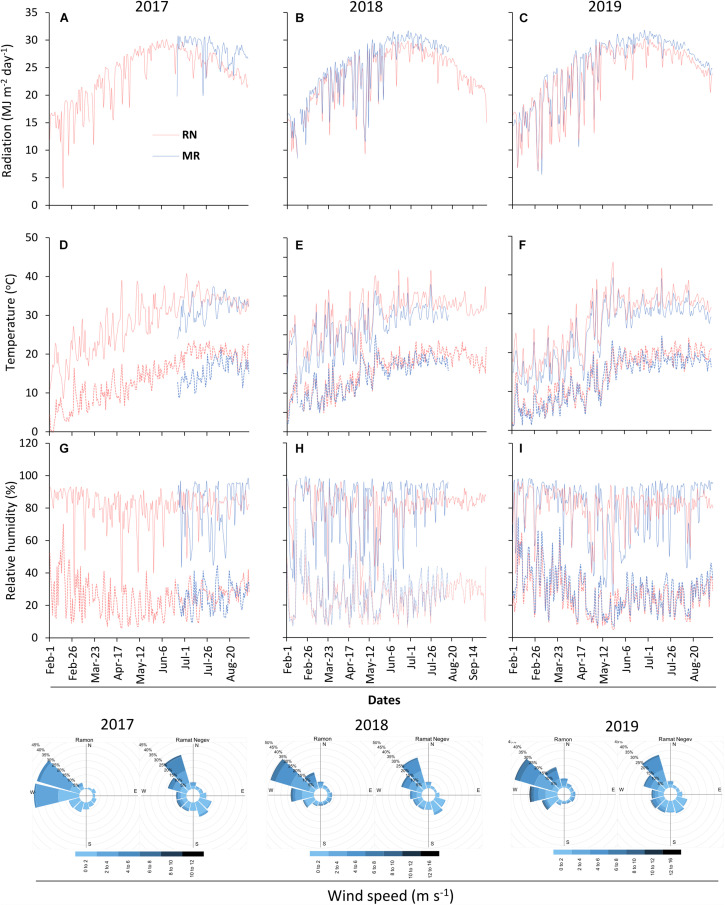
Meteorological conditions at the experimental sites. Radiation **(A–C)**, temperature **(D–F)**, relative humidity **(G–I)**, and wind speed were continuously measured at 2 m above canopy during 2017–2019 seasons. The solid and dotted lines in the temperature and relative humidity graphs denote maximum and minimum measurements, respectively. RN, Ramat Negev; MR, Ramon. The 2017 data at Ramon vineyard are from 23^rd^ May 2017 until harvest end.

**FIGURE 3:**
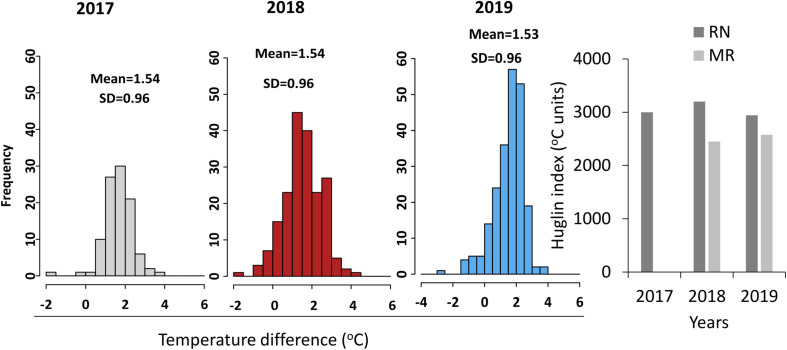
Temperature and Huglin index differences between the sites. Histograms show the frequency distribution of mean daily temperature difference between Ramat Negev (RN) and Ramon (MR) (Ramat Negev-Ramon) vineyards in 2017 (gray), 2018 (red), and 2019 (blue) seasons. SD, standard deviation. The bar graph represents the Huglin index (HI) calculated from March to August. The temperature differences between the vineyards in the 2017 season were performed using the temperature data measured from 23^rd^ May until the last harvest date 29th August. The 2017 HI of MR was not presented as the data were not available throughout the season.

The two vineyards differed in their climatic conditions (Figures 2, 3). MR vineyard experienced slightly higher incoming solar irradiance, lower temperature, both maximum and minimum, and as a result also slightly higher relative humidity. Wind speed in both vineyards is within the same magnitude range, but a slight difference in wind direction exists—the prevailing direction in MR is west-northwest, while in RN it is north-northwest. In both sites, the wind originates from the sea breeze from the Mediterranean Sea and peaks in the afternoon. The average HI computed from the meteorological data measured in each season categorized RN and MR vineyards as hot (HI > 3,000°C units) and warm (HI > 2400°C units) regions, respectively (Figure 3). These differences stem from a consistent 1.5°C difference in the daily mean temperature measured throughout the three seasons (Figure 3). Having said that, 2018’s temperature regime, especially during the spring, was warmer in both vineyards compared to 2017 and 2019 (Figures 2D–F).

### The Interaction Between Climate and Season Strongly Affected the Timing of Phenological Stages in Red and White Grapevine Cultivars

**FIGURE 4:**
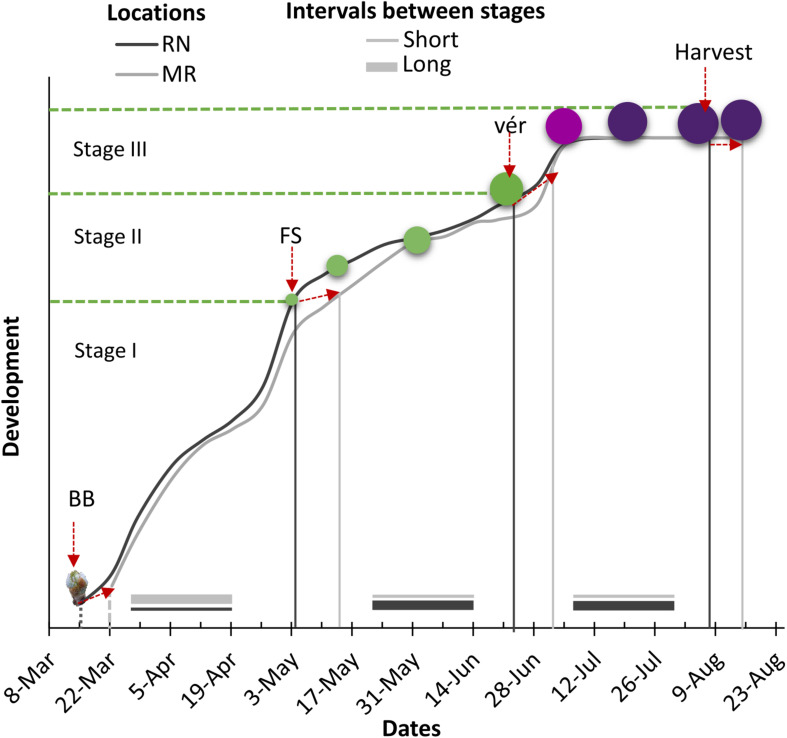
Schematic presentation of the effect of location on timing of major phenological stages and the intervals between stages for white and red cultivars. RN, Ramat Negev; MR, Ramon; BB, bud break; FS, fruit set; Vér, véraison. Stage I: vegetative growth, stage II: fruit cell enlargement and fruit hardening, stage III: fruit ripening.

The timing of phenological events was strongly affected by cultivar, site, year, and by the interaction between these factors (Supplementary Tables 3, 4). Generally, bud break (BB), fruit set (FS), and véraison (Vér) occurred earlier in the warmer RN than in MR (Figure 4 and Table 1). On average, the harvest date of the white cultivars shifted by 6–14 days from RN to MR. However, among the red cultivars, the harvest date varied more between seasons than between locations (Table 1).

**TABLE 1:** The average occurrence of phenological phases (expressed in dates) and the length between phenological phases (expressed in days) in red and white cultivars grown at Ramon (MR) and Ramat Negev (RN) vineyards from 2017 to 2019.

Cultivar	Years	Location	The timing of phenological events (dates)				The intervals between phenological phases (days)		
			Bud break (BB)	Fruit set (FS)	Véraison (Vér)	Harvest (Har)	BB to FS	FS to Vér	Vér to Har
Red	2017	RN	22-Mar ± 0.3^B^*	04-May ± 0.3^B^*	24-Jun ± 0.7^B^*	09-Aug ± 1.3B	43.2 ± 0.4^B^	50.5 ± 0.7^B^*	46.5 ± 1.0^A^*
	2018		11-Mar ± 0.3^A^*	25-Apr ± 0.4^A^*	14-Jun ± 0.6^A^*	24-Jul ± 1.2^A^*	45.3 ± 0.4^A^	49.6 ± 0.5B	40.2 ± 1.0^B^
	2019		30-Mar	13-May ± 0.2^C^*	04-Jul ± 0.4^C^*	17-Aug ± 1.1C	44	52.7 ± 0.4^A^*	43.8 ± 1.0^A^*
	2017	MR	31-Mar ± 0.4^b^	20-May ± 0.6^b^	06-Jul ± 0.5^b^	12-Aug ± 0.8^b^	50.7 ± 0.5^a^*	46.5 ± 0.6^b^	35.9 ± 0.7^b^
	2018		16-Mar ± 0.4^a^	02-May ± 0.5^a^	20-Jun ± 0.4^a^	01-Aug ± 0.9^a^	47.3 ± 0.4^b^*	48.9 ± 0.5^a^	41.8 ± 0.8^a^
	2019		07-Apr	20-May ± 0.2^b^	06-Jul ± 0.4^b^	16-Aug ± 0.7^c^	43	46.7 ± 0.3^b^	40.9 ± 0.6^a^
White	2017	RN	20-Mar ± 0.7^B^*	02-May ± 0.6^B^*	24-Jun ± 1.1^B^*	21-Jul ± 2.0^B^*	43.6 ± 0.4	52.7 ± 0.7^A^*	27.6 ± 1.6^A^*
	2018		08-Mar ± 0.6^A^*	22-Apr ± 0.8^A^*	12-Jun ± 0.9^A^*	11-Jul ± 1.2A	44.7 ± 0.5	48.4 ± 0.5^B^	28.7 ± 0.6^A^*
	2019		28-Mar	10-May ± 0.5^C^*	29-Jun ± 0.5^C^*	27-Jul ± 1.2^B^*	43	50.0 ± 0.4^B^*	28.0 ± 0.9^A^
	2017	MR	29-Mar ± 0.4^b^	17-May ± 0.8^b^	07-Jul ± 4.1c	04-Aug ± 1.1^b^	49.3 ± 0.6*	50.5 ± 0.7^a^	22.7 ± 0.9^b^
	2018		15-Mar ± 0.6^a^	30-Apr ± 0.8a	18-Jun ± 0.6^a^	15-Jul ± 0.8^a^	46.3 ± 0.5*	49.1 ± 0.6^a^	27.0 ± 0.5^a^
	2019		04-Apr	20-May ± 0.4b	5-Jul ± 0.3^b^	2-Aug ± 1.3^b^	46	46.0 ± 0.3^b^	27.5 ± 1.3^a^

Data are the overall mean value ± SE of red (n = 4 replicate × 20 cultivars) and white (n = 4 replicates × 10 cultivars) cultivars in 2017, 2018, and 2019 season. *indicates a significant difference between locations within the same season. a, b, and c indicate significant differences between the seasons at Ramon vineyard. A, B, and C indicate significant differences between seasons at Ramat Negev vineyard. BB and BB to FS in 2019 were not recorded in varietal resolution.

**FIGURE 5:**
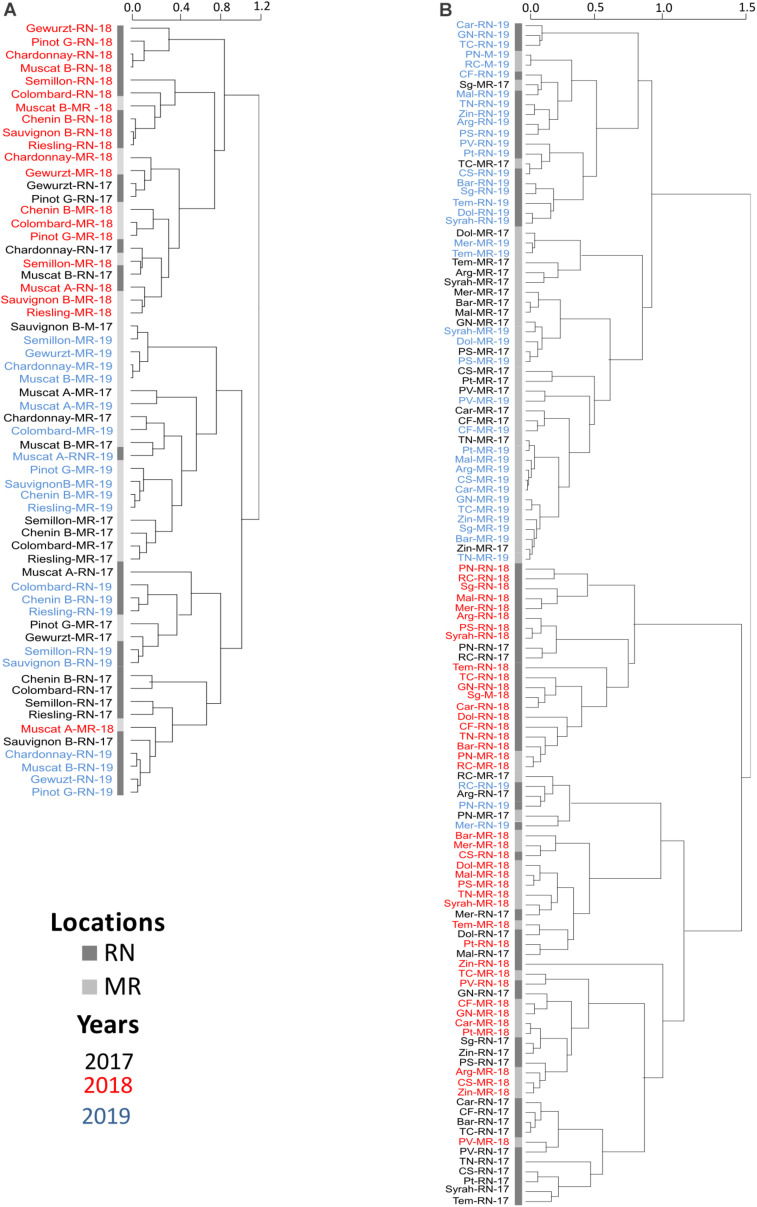
Hierarchical clustering of phenological events of white **(A)** and red **(B)** cultivars. MR, Ramon vineyard; RN, Ramat Negev vineyard. The hierarchical clustering was generated using the mean value of four replicates following normalization to the median of each phenological event on all cultivars. Cultivar names are composed by vineyard abbreviations followed by vintage (17, 18, or 19). Black, red, and blue indicate cultivars grown in 2017, in 2018, and in 2019, respectively. The number of days after February 1^st^ until the onset of each phenological stage was calculated, and the length in days was used to perform hierarchical clustering See Supplementary Table 1.

In 2018, significant phenological shifts were evident compared to 2017 and 2019 regardless of the vineyard location (Figure 5 and Table 1). These inter-seasonal differences were mainly attributed to an earlier BB in 2018 (Table 1), due to exceptionally high spring temperatures (Figure 2E). Within each seasonal cluster, cultivars were grouped by ‘location’ (Figure 5). As expected, cultivars generally considered early- and late-maturing were separated within each location cluster. For example, early maturing white cultivars, such as Chardonnay, Pinot Gris, Gewurztraminer, and Muscat Blanc, were grouped away from the late-maturing cultivars Chenin Blanc, Colombard, Riesling, and Muscat Alexandria. Similarly, among red cultivars, Pinot Noir and Ruby Cabernet grouped together within each cluster, displaying a similar phenological pattern across seasons, with earlier véraison and harvest dates. Note that the coefficient of variation of red cultivars in RN was greater than in MR, showing greater plasticity in phenology between seasons, compared with the white cultivars that displayed greater variation at MR vineyard (Figure 5 and Supplementary Figure 1 and Supplementary Table 1).

**FIGURE 6:**
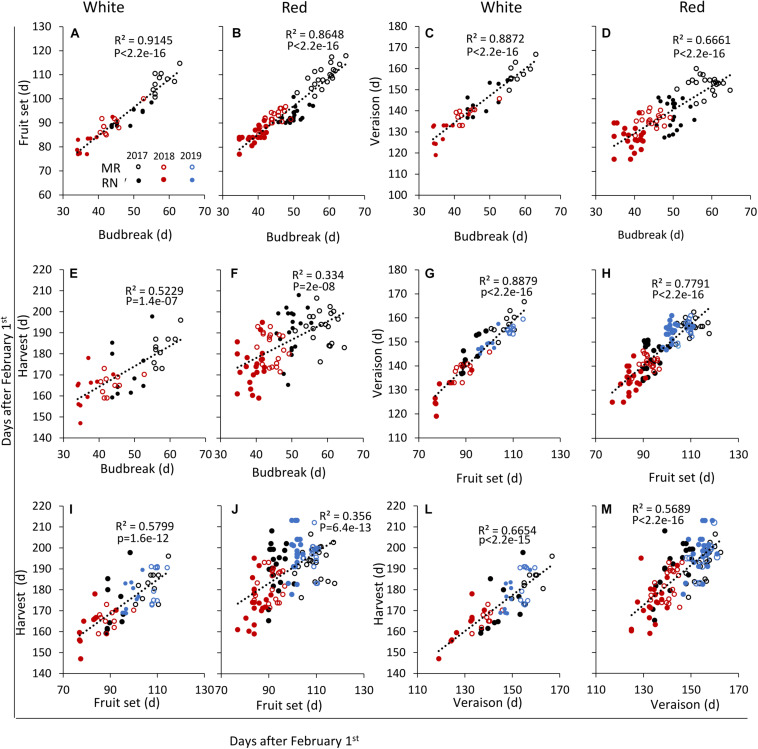
Linear regressions between the timing of various pairs of phenological events in white and red wine grapevine cultivars, respectively, as follows: fruit set to budbreak, **(A,B)**; véraison to budbreak, **(C,D)**; harvest to budbreak, **(E,F)**; véraison to fruit set, **(G,H)**; harvest to fruit set, **(I,J)**; and, harvest to véraison, **(L,M)**. In each season, data are the average values of four biological replicates (each consisting of eight–nine plants) at Ramon (MR) and Ramat Negev (RN) vineyards. (d), days after February 1^st^ until the onset of each phenological event. Open and close circles denote Ramon and Ramat Negev vineyard, respectively. Black, red, and blue indicate cultivars grown in 2017, in 2018, and in 2019, respectively. See Supplementary Table 2.

For each cultivar and location, the onset dates of the four phenological stages were correlated with each other over the 3 years of experiment (Figure 6); the onset of each stage was strongly correlated with the preceding one (Figure 6 and Supplementary Table 2). Thus, bud break and fruit set displayed particularly strong correlations in both white and red cultivars: early or late bud break onset was corresponded by early or late onset of fruit set, respectively (Figures 6A,B). In a similar way, the onsets of fruit set and véraison were strongly correlated, although this relationship was weaker among the red cultivars (Figures 6G,H). In contrast, the linkage between the onsets of the consecutive véraison and harvest stages was much less pronounced, particularly among the red cultivars (Figures 6L,M). Exceptions to the strong linkage between consecutive stages were Temperanillo, Tinta Cao, and Touriga Nacional, among the red cultivars, and Sauvignon Blanc and Semillon—among the white ones (Supplementary Table 2).

The correlations between onsets of the non-consecutive stages were generally fainter, but considerably stronger among the white cultivars. The onsets of bud break and harvest, the most departed stages, were weakly correlated among the white cultivars (Figure 6E) and quite blurry among the red ones (Figure 6F). Later in the season, the correlation between the onsets of bud break and véraison remained strong among the white cultivars (Figure 6C), but began to fold in the red group (Figure 6D). Nevertheless, the frailest relationships occurred between the onsets of fruit set and harvest, which were still positive and valid (*p* = 1.6e–12) among the white cultivars (Figure 6I), but weak in the red group (Figure 6J). This dissection of the phenological course points to the fruit ripening phase, between véraison and harvest, as the main source of variation between cultivars, particularly in the red ones (Figure 6M).

### Varietal-Specific Differences in the Duration of Phenological Phases Reflect Genotype vs. Environment Interaction in Response to the Temperature Shift Between Locations

**FIGURE 7:**
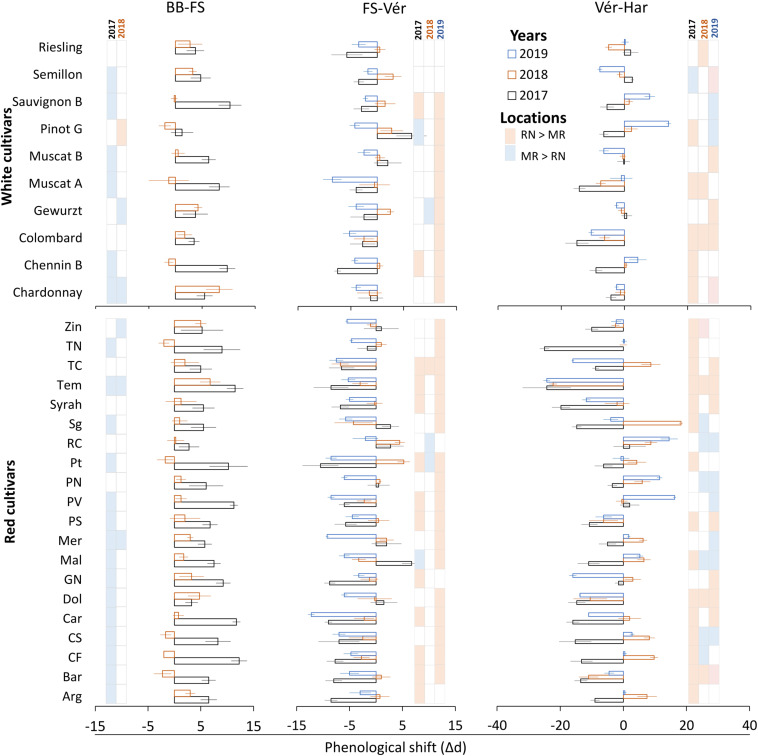
Differences between vineyards in the duration of phenological phases in white and red grapevine cultivars during 2017, 2018, and 2019 growing seasons. Calculated by subtraction of a given phase duration at Ramon (MR) from the respective phase at Ramat Negev (RN). Red and blue colors inside the grid represent significantly longer intervals at Ramat Negev and Ramon, respectively. Error bars are standard error (*n* = 4); d, days; BB, bud break; Fs, fruit set; Vér, véraison; Har, harvest. The data for BB-FS intervals are only from the 2017 and 2018 seasons (see Table 2).

Statistical analysis of the duration of the phenological phases revealed significant effects of cultivar (C), location (L), year (Y), and the interactions among them for all phases except for the period from bud break to fruit set, which was not affected by the C × Y interaction (Supplementary Tables 3, 4). In order to evaluate the effect of the location climate on the duration of phenological phases, we introduced the phenological shift. This measure was calculated by subtracting the number of days of a given phenological phase in RN from that in MR (Figure 7).

The vegetative phase, from bud break to fruit set, varied considerably among cultivars, with short periods of 40 days (Muscat Blanc, Petit Verdot, Pinot Noir, and Tinta Cao) compared to much longer ones of 57 days (Pinotage) (Table 2). The phenological shift of the vegetative phase was consistently positive and longer at MR vineyard (Figure 7); however, it was strongly season dependent, as indicated by the significant L × Y interaction (Supplementary Tables 3, 4). Comparing seasons 2017 and 2018 (BB onset on 2019 was not recorded in varietal resolution), the overall mean phenological shift among white and red cultivars was three and six-fold greater in 2017, respectively, compared to 2018.

**TABLE 2:** The duration of intervals between phenological events in red and white cultivars grown at Ramon (MR) and Ramat Negev (RN) vineyards during 2017, 2018, and 2019 seasons.

Cultivar	Bud break to fruit set interval				Fruit set to véraison interval						Véraison to harvest					
	2017		2018		2017		2018		2019		2017		2018		2019	
	MR	RN	MR	RN	MR	RN	MR	RN	MR	RN	MR	RN	MR	RN	MR	RN
**Red**																
Arg	54.5*	48.0	50.3	47.3	41.5	50.0*^A^	45.8	45.0^B^	46.3	49.3^AB^	26.5^c^	35.5*	46.5^a^	39.0	42.3^b^	41.8
Bar	47.5*	41.0^B^	43.8	46.0^A^	50.8	58.8*^A^	53.3	52.3^B^	50.0	55.0*^AB^	35.3^b^	48.8*^A^	27.5^c^	38.5*^B^	41.0^a^	45.5*^AB^
CF	55.3*^a^	43.0^B^	47.8^b^	48.0^A^	51.3	59.0*	53.3	56.0	51.0	55.8*	34.3^b^	47.5*^A^	41.0*^a^	31.3^B^	34.5^b^	34.0^B^
CS	51.3	43.0^B^	49.5	48.8^A^	42.3	49.3	46.3	48.8	45.5	52.5*	39.0^c^	54.3*^A^	45.8*^a^	37.5^B^	42.8*^b^	40.0^B^
Car	54.3*^a^	42.5^B^	50.0^b^	49.3^A^	48.0	57.0*^A^	48.5	49.3^B^	45.5	57.8*^A^	39.5^c^	55.5*^A^	48.8^a^	46.8^B^	43.0^b^	54.0*^A^
Dol	48.8	44.0	48.8	44.0	42.0	42.0^B^	43.3	43.5^B^	45.8	51.8*^A^	31.3	46.0*	36.0	46.5*	36.3	50.0*
GN	54.5*^a^	45.3	49.8^b^	46.5	48.3	57.0*^A^	52.5	53.8^B^	49.8	53.0^B^	35.5^b^	37.0^B^	43.5^a^	40.5^B^	42.0^a^	58.0^A^*
Mal	49.5*	42.0	47.3	45.5	50.0*	43.3^B^	47.8	51.0^A^	46.8	52.8*^A^	35.3^b^	46.3*^A^	34.8*^b^	28.3^C^	43.0*^a^	37.8^B^
Mer	50.0*^a^	44.3	46.3*^b^	43.3	49.0^a^	47.0^B^	50.8^a^	48.8^AB^	43.8^b^	53.0*^A^	34.3	39.3^A^	32.5	26.3^B^	29.3*	27.5^B^
PS	49.8*	43.0	49.3	47.3	43.5	49.3*	46.0	43.5	44.3	48.8*	38.0	48.8*	34.0	40.3	39.8	46.0*
PV	50.8*^a^	39.5^B^	46.0^b^	44.8^A^	52.5^b^	58.5*	56.3^a^	58.5	50.5^b^	59.0*	45.3^b^	43.3^A^	45.8^b^	46.3^A^	52.3*^a^	36.0^B^
PN	45.5	39.5	43.5	42.3	45.3^ab^	44.8^B^	47.8^a^	48.0^A^	43.5^b^	49.5*^A^	27.3^b^	30.8^B^	42.0*^a^	36.0^A^	46.8*^a^	35.3^A^
Pt	56.5*^a^	46.3	47.5^b^	49.3	40.8^c^	51.3*^A^	49.8^a^*	44.5^B^	45.5^b^	54.0*^A^	43.5	49.8*^A^	46.8	42.5^B^	43.3	44.0^AB^
RC	45.0	42.3	43.0	42.8	47.0	44.3	48.8*	44.3	45.0	47.0	38.5	36.5	44.0*	35.3	45.5*	31.0
Sg	50.0*^a^	44.5	45.0^b^	44.0	51.5	48.8^B^	49.5	51.8*^A^	47.8	53.5*^A^	35.0^c^	49.8*^A^	53.8*^a^	49.5^B^	44.3^b^	48.3^A^
Syrah	50.8	45.3	49.8	48.5	44.0	50.8*	46.5	46.8	46.5	51.5*	28.0^b^	47.8*^A^	34.5^ab^	36.5^B^	37.5^a^	49.3*^A^
Temp	53.0*	41.5	49.0*	42.3	35.8^b^	44.3*	42.0^a^	45.0	41.8^a^	47.0*	28.5^b^	52.8*	42.8^a^	65.0*	30.8^b^	55.0*
TC	49.3	44.3^B^	50.5	48.5^A^	53.5	60.0*^A^	50.0	56.8^B^*	48.8	56.3*^B^	39.0^b^	47.8*^B^	53.8^a^	45.0^ B^	41.0^b^	57.0*^A^
TN	49.8*^a^	39.3	42.3^b^	43.7	45.0^b^	47.7^B^	50.8^a^	50.0^AB^	48.3^ab^	53.0*^A^	42.8^a^	68.0*^A^	37.8^b^	38.0^B^	41.0^ab^	40.7^B^
Zin	47.5	42.3	47.3*	42.3	48.5	47.5^C^	48.0	49.0^B^	47.5	53.0*^A^	41.3	51.3*^A^	44.8	47.3*^B^	41.3	43.5^C^
**White**																
Chardonnay	50.0*	44.5	50.8*	42.5	49.0	50.3	45.3	45.5	47.0	51.0*	17.5^b^	21.8*^B^	25.0^a^	26.3^A^	18.5^b^	21.0*^B^
WhiteChenin B	55.0*^a^	45.3^B^	47.5^b^	48.8^A^	49.8^a^	57.3*^A^	50.5^a^	50.0^B^	45.5^b^	49.8*^B^	25.8^b^	34.8*	33.3^a^	32.8	35.5^a^	31.3
Colombard	48.8	45.3	46.5	44.8	49.0^ab^	51.8	52.3^a^	52.5	45.5^b^	50.8*	28.8	43.8*^A^	27.5	33.8*^B^	25.3	35.8*^B^
Gewurzt	48.3	44.5	47.0*	42.8	46.0	48.5^A^	44.0*	41.5^B^	45.0	49.0*^A^	23.3^ab^	22.5^B^	26.0^a^	27.0^A^	21.5^b^	24.0*^AB^
Muscat A	51.8*	43.5	47.3	48.5	52.0^a^	56.0^A^	45.8^ab^	46.3^B^	45.3^b^	53.8*^A^	30.5^ab^	44.8*^A^	24.5^b^	32.0*^B^	31.0^a^	32.0^B^
Muscat B	46.0*	39.8^B^	43.3	42.8^A^	53.0^a^	51.0	48.0^b^	47.5	47.5^b^	50.0*	21.5^ab^	21.8	26.0^a^	26.5	17.5^b^	24.0*
Pinot G	44.8	43.5^A^	43.5*	40.3^B^	54.8*	48.3^B^	52.3	49.5^AB^	47.5	51.8*^A^	17.5^c^	24.0*^B^	30.3^b^	28.0^A^	36.0*^a^	22.0^B^
Semillon	50.3*^a^	45.5	44.5^b^	46.5	54.3^a^	57.8^A^	52.5^a^	49.5^B^	46.5^b^	48.3^B^	17.5*^b^	15.0^B^	23.5^a^	25.0^A^	18.3^b^	26.0*^A^
Sauvignon B	52.8*	42.5	47.3	44.5	44.5^b^	47.5*^A^	49.8^a^	49.0^A^	43.3^b^	45.5*^B^	18.0^c^	23.5^B^	25.3^b^	23.8^B^	37.3*^a^	29.3^A^
Riesling	45.8	42.0^B^	45.5	45.8^A^	52.5	58.3^A^	50.0	49.5^B^	46.8	50.3^B^	26.3^b^	24.3^B^	28.3^b^	33.3*^A^	34.5^a^	34.3^A^

*indicates significant differences between locations within the same season. a, b, and c indicate significant differences between the seasons at Ramon vineyard. A, B, and C indicate significant differences between seasons at Ramat Negev vineyard. Data are the mean value of four replicates (n = 4). The data of bud break to fruit set are from 2017 and 2018.

Among the white cultivars, Sauvignon Blanc, Chenin Blanc, Muscat of Alexandria, and Muscat Blanc displayed strong phenological shifts only in 2017 (6–10 days; Figure 7). In contrast, Pinot Gris, Semillon, Gewurztraminer, Riesling, and Colombard exhibited consistent moderate shifts (1–4 days), while Chardonnay showed large shifts in both years (Figure 7). Among the red cultivars, Tinta Cao, Pinotage, Petit Verdot, Carignan, Cabernet Sauvignon, Cabernet Franc, and Barbera displayed large shifts in 2017, but negligible ones in 2018. Merlot and Tempranillo were severely affected in both years, contrary to Pinot Noir, Ruby Cabernet, and Syrah that were unaffected. Most of the other red cultivars exhibited mild to moderate shifts, with considerable differences between seasons (Figure 7).

In contrast to the vegetative phase, phenological shifts of the fruit growth phase (FS-Vér) were primarily negative; this phase was shorter at MR vineyard (2017 and 2019 seasons; *P* < 0.001), with very few exceptions among cultivars (no significant shifts were recorded in 2018, Figure 7 and Table 1). Among white cultivars, Chenin Blanc, Semillon, and Riesling displayed the longest phase (57–58 days) at RN in 2017, and Gewurztraminer showed the shortest period in 2018 at MR (Table 2). Among the red cultivars, Tempranillo exhibited the shortest FS-Ver (35 days) in 2017, and Tinta Cao the longest (62 days) FS-Vér in 2018 at MR vineyard (Table 2). Gewurztraminer and Pinot Gris among the white cultivars and Ruby Cabernet, Pinotage, and Malbec among the red cultivars exhibited significantly contrasting trends of phenological shifts between the seasons (Figure 7). Pinotage, Carignan, and Cabernet Sauvignon displayed the strongest shifts in FS-Vér among the red cultivars.

The duration of Vér-Har phase varied from 23 to 29 days in the white and from 36 to 47 days in the red cultivars (Table 1). The end of this phase was defined upon obtaining target Brix values, 20±1% and 23±1% for white and red cultivars, respectively. Noteworthy, however, is the failure of several cultivars to meet this threshold, which depended on the location and season. Cultivars with particular susceptibility were Pinot Noir, Barbera, Dolcetto, Tempranillo, and Zinfandel; the Brix of which failed to increase beyond a certain value, or furthermore, most of the fruit shriveled before reaching harvest. Similar to fruit development, the phenological shift of the Vér-Har phase was mostly negative, indicating an extension of this period in the warmer RN compared to MR, with considerable differences between seasons (Table 2). Among the white cultivars, Colombard and Muscat of Alexandria displayed the strongest shifts (up to 15 days); Chardonnay, Gewurztraminer, Muscat Blanc, Semillon, and Riesling hardly responded; Semillon, Pinot Gris, and Chenin Blanc showed inconsistent phenological shifts that varied between years (Figure 7). Chardonnay, Gewurztraminer, Muscat Blanc, and Semillon had the shortest ripening periods (18 to 27 days, depending on the season), whereas Chenin Blanc, Colombard, Muscat Alexandria, and Riesling displayed much longer ripening periods (26 to 45 days) (Table 2). In an effort to identify sensitive cultivars to seasonal variation, we calculated the coefficient of variance (CV) for each cultivar separately at each site across three seasons (Supplementary Figure 2). The CV can provide insights into the effect of environmental variability on cultivar sensitivity. Here, the higher the CV, the greater the sensitivity of a given cultivar for the respective trait in response to seasonal variation (Reed et al., 2002). The CV analysis for Vér-Har phases revealed that Muscat Blanc and Pinot Gris in MR and Muscat Alexandria and Semillon in RN were the most responsive cultivars to seasonal differences (Supplementary Figure 2).

The negative phenological shift of the Vér-Har phase was, on average, much stronger among the red cultivars, and considerable variability was monitored between cultivars and seasons (Figure 7). Tempranillo, Dolcetto, Syrah, and Barbera displayed consistent and strong negative phenological shifts, whereas Ruby Cabernet, Pinot Noir, and Petit Verdot showed positive shifts. Zinfandel, Pinotage, and Merlot were hardly influenced by the location, as indicated by very small shifts. In contrast, quite many red cultivars exhibited seasonal variability in the direction and strength of the phenological shift (Figure 7). CV analysis among the red cultivars revealed that Argaman and Pinot Noir in MR and Grenache Noir, Malbec, Merlot, Cabernet Sauvignon, and Cabernet Franc in RN were the most sensitive to differences between seasons (Supplementary Figure 2).

### The Greatest Differences Between Vineyard Locations in Fruit Organic Acids Were Observed at Véraison and Their Level of Variance Differed Between Seasons

**FIGURE 8:**
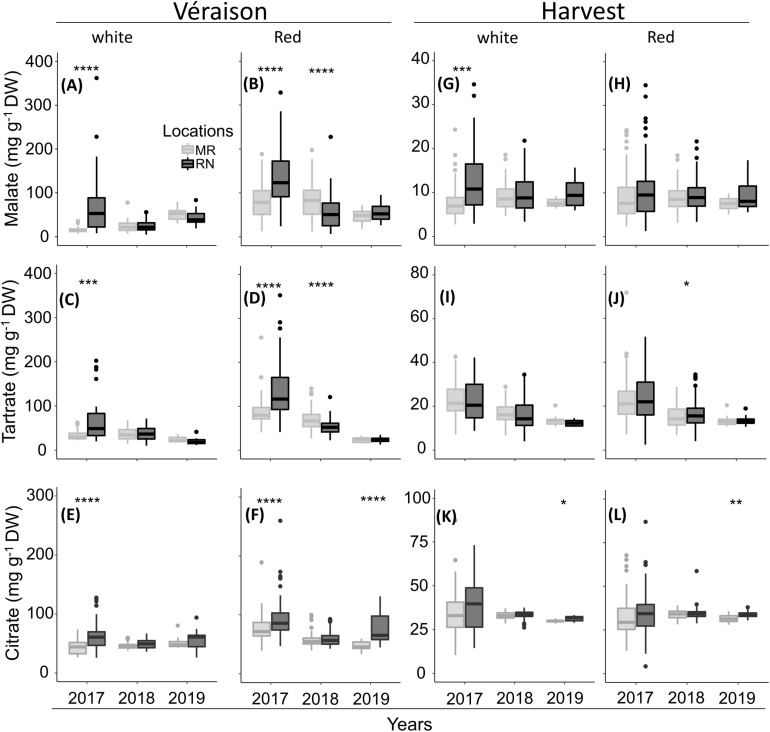
The pulp organic acids at véraison **(A–F)** and harvest **(G–L)** in white **(A,C,E** and **G,I,K)** and red **(B,D,F** and **H,J,L)** cultivars grown at Ramon (MR) and Ramat Negev (RN) vineyards from 2017 to 2019. Data for 2017 and 2018 are the average value across all white (*n* = 4 replicates × 10 cultivars) and red (*n* = 4 replicates × 20 cultivars) cultivars at Ramon and Ramat Negev vineyards. Data of 2019 are the average value across all white (*n* = bulked replicate × 10 cultivars) and red (*n* = bulked replicate × 20 cultivars) cultivars at Ramon and Ramat Negev. Box limits are first and third quartile. Boxplots followed by asterisks indicate significant difference at *P < 0.05, **P < 0.01, ***P < 0.001, and ****P < 0.0001 level between vineyard locations within the same year based on non-parametric *t*-test (see Supplementary Tables 6, 7).

**FIGURE 9:**
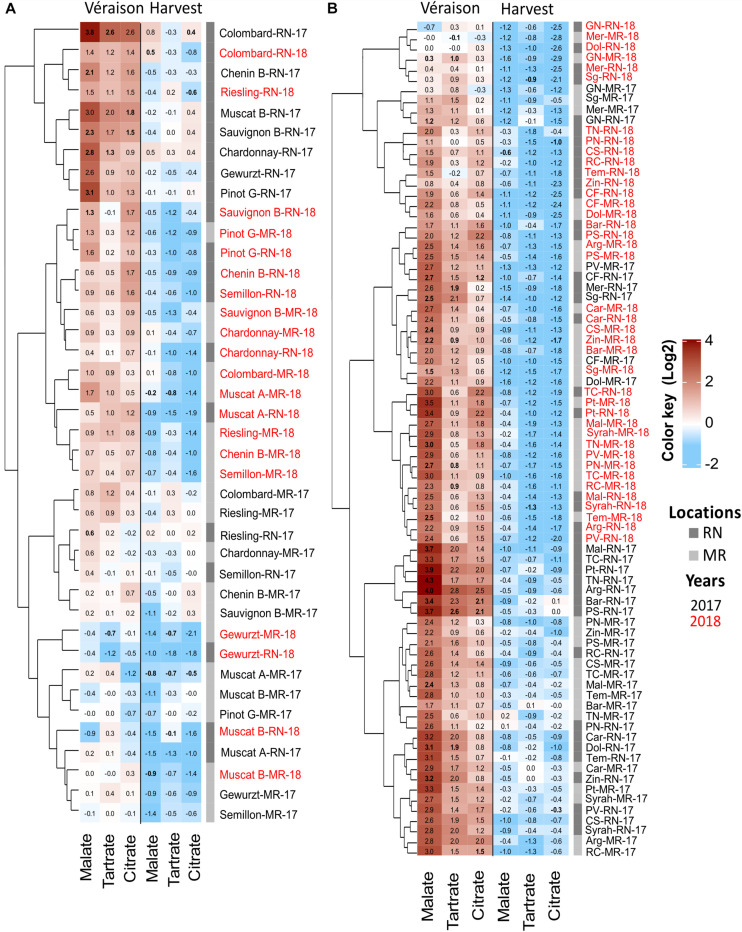
Heatmap of pulp organic acids in white **(A)** and red **(B)** grapes. The heatmap was generated using the mean value of four biological replicates following normalization to the median of each metabolite on all cultivars and log2 transformation. Cultivar names are composed by vineyard abbreviations (MR and RN) followed by vintage (17 or 18). Cultivar names with black and red color indicate samples collected in 2017 and 2018, respectively. MR, Ramon; RN, Ramat Negev. Red and blue rectangles represent an increase and decrease of metabolite relative to the median (see Supplementary Material 1 and Supplementary Tables 6, 7).

The change in malate, tartrate, and citrate was seasonal and cultivar dependent (Figures 8, 9). The greatest differences were observed in 2017, when, at véraison, higher OA were measured at RN compared to MR, but no marked differences between locations were scored at harvest; the higher content of malate in white berries at RN was an exception among OA (Figure 8). In 2018 and 2019 seasons, OA in white berries, at both véraison and harvest, were not affected by location, excluding citrate at harvest in 2019, which was higher at RN (Figure 8K). In contrast, malate and tartrate levels were considerably high at MR compared to RN in 2018 in red berries at véraison, but no marked differences between locations were identified at harvest in all seasons. At harvest, tartrate (in 2018) and citrate (in 2019) in red berries were exceptionally high at RN (Figure 8). Hierarchical clustering of 2017 and 2018 data highlighted the segregation not only between the seasons but also between vineyards, particularly for white cultivars (Figures 9A,B). In 2017, white cultivars at RN separated from MR, with the exception of Muscat Alexandria, Riesling, and Semillon, cultivars with low acid accumulation at véraison and similar to the levels measured at MR vineyard (Figure 9A). Red cultivars exhibited considerable plasticity at véraison in their OA concentrations that varied substantially between seasons, with the exception of Grenache Noir, Sangiovese, Merlot, Petit Verdot, Cabernet Franc, and Dolcetto, whereas location-clusters were clearly discerned within each season-cluster (Figure 9B).

The pace of OA decrease from véraison to harvest considerably varied between vineyards and between seasons in each vineyard (Figure 9 and Supplementary Table 5). For example, in 2017, the average OA reduction in red berries was almost twice greater at the warmer RN compared to MR (Supplementary Table 5). OA reduction was significantly moderate in the white cultivars compared to the reds at RN. Comparing vineyards, the overall OA reduction among white cultivars at RN was greater than at MR. On the contrary, in 2018, the reduction of malate and tartrate in red berries was smaller at RN than at MR, whereas no differences between locations were observed in the white cultivars. Generally, cultivars grown at MR exhibited lower pulp OA concentration at véraison and reached harvest with minimum loss of acidity (Figure 9 and Supplementary Table 5). Moreover, white cultivars appeared to be rigid in their OA degradation compared to the red ones.

### Pulp Sugar Differences Were Predominantly Expressed in Hexose/Sucrose Ratio and Were Largely Affected by Cultivar and Location

**FIGURE 10:**
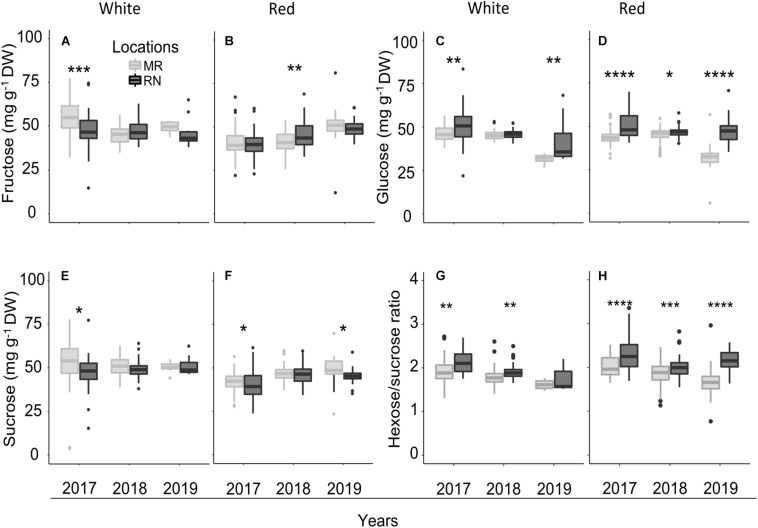
The pulp sugars (fructose, **(A,B)**; glucose, **(C,D)**; sucrose, **(E,F)**; and the hexose/sucrose ratio, **(G,H)** at véraison in white **(A,C,E,G)** and red **(B,D,F,H)** cultivars grown at Ramon (MR) and Ramat Negev (RN) vineyards from 2017 to 2019. Data for 2017 and 2018 are the average value across all white (*n* = 4 replicates × 10 cultivars) and red (*n* = 4 replicates × 20 cultivars) cultivars at Ramon and Ramat Negev vineyards. Data of 2019 are the average value across all white (*n* = bulked replicate × 10 cultivars) and red (*n* = bulked replicate × 20 cultivars) cultivars at Ramon and Ramat Negev. Box limits are the first and third quartile. Boxplots followed by asterisks indicate significant difference at *P < 0.05, **P < 0.01, ***P < 0.001, and ****P < 0.0001 level between vineyard locations within the same year based on non-parametric *t* test (see Supplementary Tables 8, 9 and Supplementary Material 1).

Statistical analysis of pulp sugars at véraison revealed significant location effects (Supplementary Figure 1), but the effect of cultivar differed between white and red berries; sugars did not differ significantly between white cultivars with exception of sucrose in 2018. The mean pulp sugars of all cultivars ranged from 32 to 54 mg g^–1^ DW, with considerable variation among cultivars and locations (Figure 10 and Supplementary Table 8). The greatest differences in pulp sugars of white cultivars were observed between locations in 2017; across all white cultivars, the average fructose and sucrose concentrations were significantly higher at MR, in contrast with glucose, which was higher at RN (Figure 10C). Notably, in 2018 and 2019 seasons, pulp sugars of white cultivars at véraison did not differ between locations, excluding glucose in 2019, which was higher at RN (Figure 10C). Hexose/sucrose ratio was significantly higher at RN vineyard in 2017 and 2018 seasons (Figure 10G) with significant cultivar × location interaction effect in 2018 (Supplementary Material 1).

Among the red cultivars at véraison, fructose was significantly affected by cultivar and location interaction (*P* < 0.0001) in 2017 (Supplementary Material 1), while sucrose showed this course both in 2017 and 2018 seasons. Fructose concentration was significantly higher at RN only in 2018 (Figure 10B), while glucose exhibited this trend throughout all three seasons (Figure 10D). In contrast, sucrose was significantly lower at RN in 2017 and 2019 (Figure 10F). Hexose/sucrose ratio at the red cultivars’ véraison was predominantly affected by cultivar and location (Supplementary Material 1). Noteworthy, this ratio was significantly higher at warmer RN than cooler MR in all seasons (Figure 10H).

**FIGURE 11:**
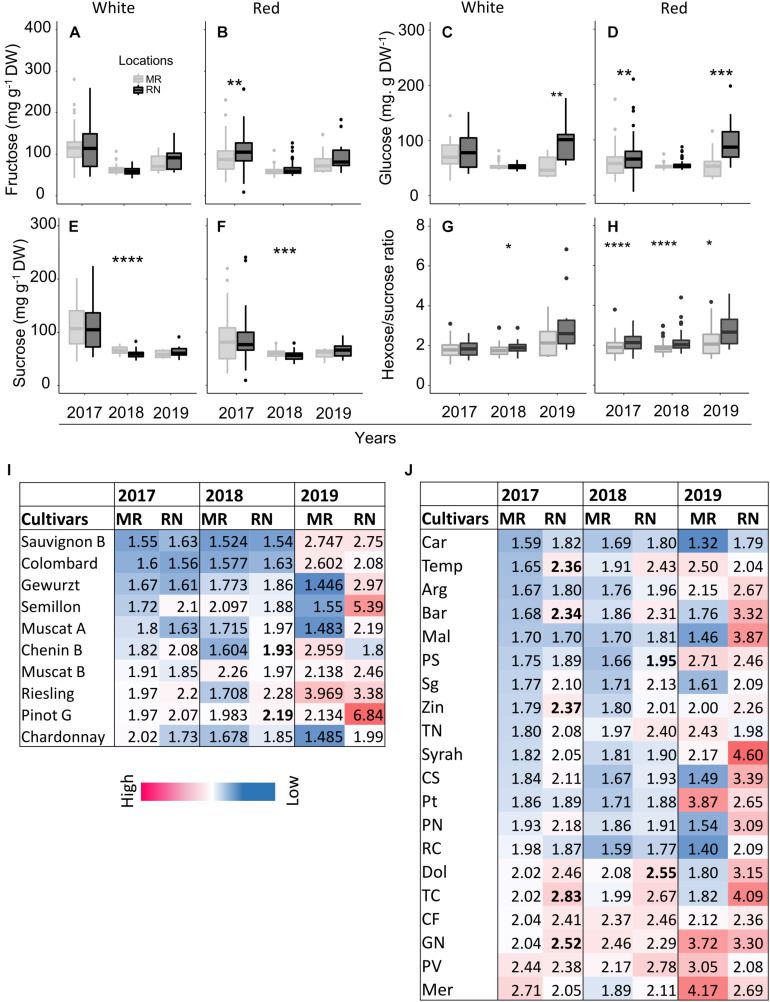
The pulp sugars **(A–F)** and hexose-to-sucrose ratio **(G–H)** at harvest in white and red cultivars grown at Ramon (MR) and Ramat Negev (RN) vineyards from 2017 to 2019. Data for 2017 and 2018 are the average value across all white (*n* = 4 replicates × 10 cultivars) and red (*n* = 4 replicates × 20 cultivars) cultivars at Ramon and Ramat Negev vineyards. Data of 2019 are the average value across all white (*n* = bulked replicate × 10 cultivars) and red (*n* = bulked replicate × 20 cultivars) cultivars at Ramon and Ramat Negev. Box limits are the first and third quartile. Boxplots followed by asterisks indicate significant difference at *P < 0.05, **P < 0.01, ***P < 0.001, and ****P < 0.0001 level between vineyard locations within the same year based on non-parametric *t* test. The mean value of hexose-to-sucrose ratios in white and red cultivars in varietal resolution are shown in heatmap **(I–J)**. Cultivar means in bold represent significant differences between locations within the cultivar. Data for 2017 and 2018 are mean of four biological replicates in each location separately; results were validated in 2019 using bulked replicate. In 2019, each cultivar’s replicates were pooled at each location (*n* = 10 or 20, for white or red cultivars, respectively).

Among white cultivars at harvest, the two-way ANOVA in each season revealed no significant effect of cultivars on pulp sugars except for sucrose in 2018, which was significantly affected by cultivars and location interaction (Supplementary Material 1). Fructose was not affected by location in all seasons (Figure 11A). However, the location had a significant effect on sugars, particularly in 2018. The average sucrose (in 2018) and glucose (in 2019) across all cultivars were significantly higher at MR and RN, respectively (Figures 11C,E), whereas hexose/sucrose ratio was significantly high only in 2018 (Figure 11G). Among white cultivars, only Chenin Blanc and Pinot Gris showed a significant difference between locations (Figure 11I).

In the red cultivars, pulp hexoses were significantly higher at RN in 2017 and 2019, but not in 2018 (Figures 11B,D), while sucrose was lower at RN in 2018 (Figure 11F). Overall, pulp sugars at harvest tended to be lower in 2018 in both red and white cultivars, compared to the two other years. Only sucrose showed significant varietal differences (*P* < 0.0001), whereas fructose and glucose were significantly affected by location (Supplementary Material 1), being consistently lower at MR vineyard (Figures 11B,D). Subsequently, the mean hexose/sucrose ratio ranged from 1.88 to 2.25, exhibiting highly significant varietal differences (*p* < 0.0001). In addition, differences between locations were highly significant (*p* < 0.0001), with higher ratios at RN (Figure 11H). Cultivars Petit Verdot, Merlot, and Grenache Noir displayed opposite conduct, as well as higher mean hexose/sucrose ratio (Figure 11J). In 2019, hexose/sucrose ratios were remarkably extreme to both ends compared to the former two years.

### Principal Component Analysis Highlights Responsive and Non-responsive Cultivars to Location and Season Differences

**FIGURE 12:**
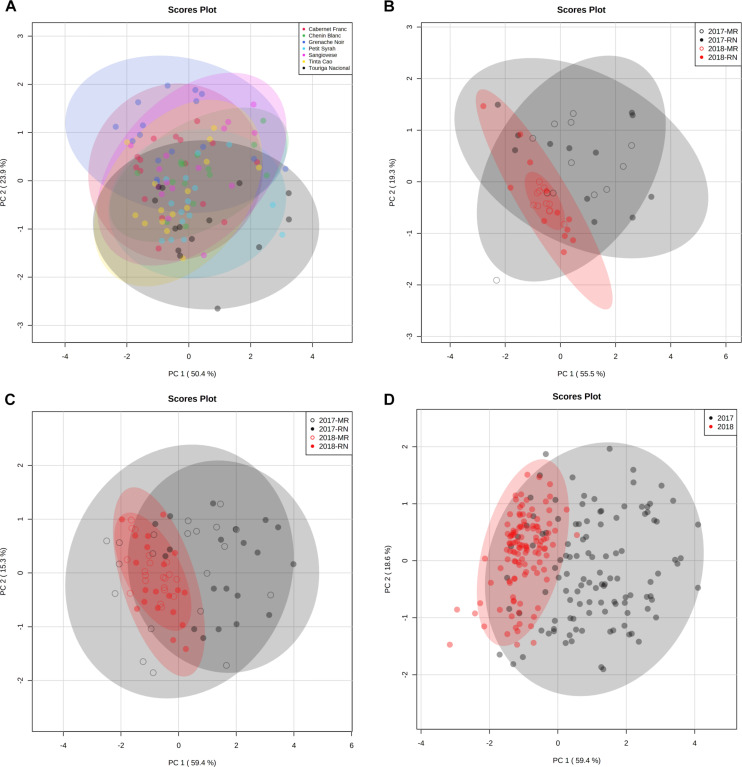
Principal component analysis (PCA) of grapevine cultivars based on berry organic acids and sugar data at harvest in 2017 and 2018 seasons. PCA was first plotted for each cultivar (data are not shown) and a two-way ANOVA model (Supplementary Material 2) was performed using PCA scores for each cultivar separately. The analysis resulted in the identification of four subsets of cultivars that were used in separate PC plots. The ellipse indicates 95% confidence region based Hotelling’s *T*^2^ test. **(A)** PCA of cultivars that were not affected by location nor by season. The set includes in the red Cabernet Franc, Grenache Noir, Petit Syrah, Sangiovese, Tinta Cao, and Touriga Nacional and in the white Chenin Blanc. **(B)** PCA of cultivars affected by location and season. The set includes in the red Ruby Cabernet and in the white Muscat Alexandria and Colombard. **(C)** PCA of cultivars affected by the interaction of location and season. The set includes in the red Barbera, Dolcetto, Petit Verdot, and Pinot Noir and in the white Riesling. **(D)** PCA of cultivars affected by season. The set includes in the red Argaman, Cabernet Sauvignon, Carignan, Malbec, Merlot, Pinotage, Syrah, Tempranillo, and Zinfandel and in the white Chardonnay, Gewurztraminer, Muscat Blanc, Pinot Gris, Semillon, and Sauvignon Blanc. The PCs were generated using the raw data of four biological replicate in 2017, 2018, and 2019 seasons following log transformation and Pareto scaling. RN, Ramat Negev; MR, Ramon.

PCs were analyzed and plotted using OA and main sugar harvest data of 2017 and 2018. A two-way ANOVA (Supplementary Material 2) was performed using PCA scores for each cultivar. The analysis resulted in four groups of cultivars: (i) cultivars not affected by location and season (non-responsive cultivars) (Supplementary Figure 3A), (ii) cultivars affected by season and location (Figure 12B), (iii) cultivars affected by location and season interaction (Figure 12C), and (iv) cultivars affected by season only (Supplementary Figure 3D). Then, four sets were plotted on PCs (Figures 12A–D).

In the non-responsive cultivars’ PCA (Figure 12A), cultivars were separated both on PC1 and PC2, due to the positive contribution of sugars (Supplementary Material 2) on PC1 and the inverse contribution of malate and citrate on PC2 (Supplementary Material 2). In PCA of cultivars affected by location and season (Figure 12B), samples were separated both on PCs, due to the positive contribution of tartrate, citrate, fructose, and sucrose on PC1 (Supplementary Material 2) and due to the inverse contribution of malate on PC2. In PCA of cultivars affected by location and season interaction (Figure 12C), 2017 and 2018 samples were separated on PC1, explaining 59.4% of the total variance due to the contribution of citrate, fructose, and sucrose (Supplementary Material 2). Malate was the major negative contributor to PC2. In season-responsive cultivars’ PCA (Figure 12D), PC1 represented 59.4% of the total variance and separated 217 samples from 2018 mainly due to the positive contribution of citrate, tartrate, fructose, and sucrose (Supplementary Material 2). Malate was the major positive contributor to PC2 (Supplementary Material 2).

## Discussion

One of the most striking patterns of phenological changes over the past two decades due to the rising temperature is the earlier onset of phenological events (Jones and Davis, 2000; Duchêne et al., 2010; van Leeuwen and Darriet, 2016; Piao et al., 2019). Mimicking this shift by setting experimental plots in two vineyards, differing in their mean daily temperature by 1.5°C, we showed earlier onset of bud break, fruit set, and véraison at the warmer RN vineyard with greater variations between the seasons in the timing of harvest than in the cooler MR vineyard (Supplementary Figure 1). Such changes might influence the véraison-harvest time-window, imposing significant consequences on berry ripening and engustment and, subsequently, on wine quality (Morales-Castilla et al., 2020), hence defining the suitability of a given cultivar to a certain region. For instance, the date of harvest among white varieties was considerably earlier in warmer RN vineyard, while red cultivars were more affected by the season, and within each group, a gradient between early and late-ripening varieties was recorded (Figure 5). Rapid phenological progress in warmer climates might provide better yield quality for many white cultivars (e.g., Chenin Blanc), shifting bloom and véraison earlier to cooler months, thus shortening the exposure of the fruit-ripening phase to prevent potentially detrimental heat effects during the summer. The situation was more complex, however, with the red cultivars, most of which displayed a longer duration of the fruit-ripening phase, required to reach a higher Brix threshold and simultaneously accomplish the desired engustment. In temperate climates, a shift to earlier fruit set and development might bring the fruit-ripening phase directly into the warmest summer period (Sadras and Moran, 2013), which is clearly disadvantageous.

The relationships between the seasonal course and berry phenology is a key element determining fruit quality in a given year. For instance, vintage was shown to be a predominant factor affecting grape and wine composition of Cabernet Sauvignon and Shiraz berry (Antalick et al., 2020); in Merlot, the metabolic response to post-véraison water deficit was consistently affected by interseason weather variability (Herrera et al., 2017). In the present study, interseasonal variability had a significant effect on the timing of phenological events; 2018 data were clearly different from those of 2017 and 2019 (Figure 5), mainly due to (i) earlier bud break, an outcome of a warmer winter and earlier spring and (ii) the correlation shown between the onsets of bud break, véraison, and harvest (Figure 6). These results differ from Ruml et al. (2016), who conducted a long-term study of 20 cultivars in Serbia and reported that shifts of berry ripening into warmer conditions resulted from earlier bloom and véraison rather than from the onset of bud break. Nevertheless, the onset of bud break appears exceedingly critical to the time course of berry development and ripening, particularly in arid regions, where year-to-year variations in the winter-spring interphase are very common. Interestingly, the analysis of coefficient of variance revealed resilient cultivars to the seasonal variation, including Tempranillo and Tinta Cao at MR, Petit Verdot at RN, and Semillon at both sites (Supplementary Figure 1).

### The Duration of Berry Development and Ripening Phases Were Extended at the Warmer RN Vineyard

Despite extensive research on grapevine phenology, only a few studies have focused on the interrelations between the onsets of phenological phases (Jones and Davis, 2000; Gladstones, 2011; Tomasi et al., 2011; Bock et al., 2014; Ruml et al., 2016). Climate conditions might lead to substantial asynchrony during development. For instance, heat treatments right after fruit set (at the fruit herbaceous stage) delayed the onset of véraison (Lecourieux et al., 2017). Furthermore, weather events and characteristics of each phenological phase have important consequences on berry development. Hence, the duration of each phase should be considered (Jones and Davis, 2000). An example of the climate effect on phenological intervals is the vegetative phase from bud break to fruit set, which is susceptible in the temperate regions to frost and hailstorm (Davenport et al., 2008) and to heatwaves in Mediterranean regions (Webb et al., 2010; Arrizabalaga-Arriazu et al., 2020). The longer the period, the higher the chances to incur into environmental constraints. This interval is usually shorter under high temperatures due to a rapid phenological pace (Tomasi et al., 2011). Therefore, a shorter vegetative phase was expected at the warmer RN vineyard. This was confirmed for all white and red cultivars, with exception of Colombard and Riesling among whites and Pinot Noir, Ruby Cabernet, Syrah, and Tinta Cao, which were not affected by the location (Figure 7).

### A Prolonged Pre-véraison Interval Can Expose the Cluster to Recurrent Heatwaves

Gladstones (2011) emphasized the susceptibility of grapevine berries to excessively high temperatures during the fruit growth phase, from fruit set to véraison. Direct exposure of clusters to sunlight was shown to reduce methoxypyrazine accumulation by 21–44% (Ryona et al., 2008). Excessive heat decreased malate and increased concentrations of amino acids (Gouot et al., 2019), many of which participate in wine aroma biosynthesis (Garde-Cerdán and Ancín-Azpilicueta, 2008; Gutiérrez-Gamboa et al., 2020). Considering the intermittent heat waves that characterize the spring in arid regions (April–May, northern hemisphere), the relative duration of this phase is assumed to significantly affect berry quality traits (Gouot et al., 2019). The longer the phase, the higher the risk of high temperature events to imbalance the accumulation of precursors for aroma and quality-related compounds, consequently affecting the final wine quality. In the present study, several cultivars displayed significantly shorter fruit growth phase, among which were Dolcetto, Petit Syrah, Pinot Noir, and Tempranillo within the red cultivars and Sauvignon Blanc among the white cultivars (Table 2). The majority of the red cultivars examined exhibited considerable extension of the fruit growth phase at the warmer RN site, with only few inconsistent exceptions. Among the white cultivars, Chardonnay, Muscat Blanc, Sauvignon Blanc, and Semillon displayed relatively small phenological shifts in this stage (Figure 7 and Table 2). A relatively rigid duration, manifested by a small phenological shift between locations, may indicate a degree of genetic resilience. Nevertheless, the direct contribution of shorter or rigid duration of the fruit growth phase to the final berry or wine quality strongly depends on the consecutive fruit-ripening phase and requires further research. In addition, it is possible to suggest that the difference in hydric behavior between cultivars may provide an explanation for the differences in berry ripening and, furthermore, in the tendency for premature dehydration (Gutiérrez-Gamboa et al., 2019). For instance, Chardonnay was reported as an anisohydric variety, while Sauvignon Blanc as an isohydric variety (Gutiérrez-Gamboa et al., 2019). Having that said, further investigation is needed on the physiology of the different varieties to draw solid conclusions.

### Post-véraison at the Warmer RN Vineyard Might Lead to Metabolic Disorders

The fruit-ripening phase, from véraison to harvest, determines the sugar/acid balance and engustment in the developing berry (Van Leeuwen et al., 2019; Morales-Castilla et al., 2020). Opposite to milder climates as Bordeaux, where longer and warmer growing seasons provide greater ripening potential (Jones and Davis, 2000), an extended fruit-ripening phase under the much higher temperature regime characterizing arid regions might lead to disorder in sugar accumulation, phenylpropanoid degradation, and sunburns (Greer and Weedon, 2013; Pastore et al., 2017). Under temperate climate regions, warmer seasons were associated with a shortened ripening phase (Tomasi et al., 2011; Alikadic et al., 2019). In the present study, the fruit-ripening phase significantly extended at the warmer RN vineyard (Table 2). This discrepancy can be easily explained in terms of an optimum temperature curve that the complex fruit-ripening process obeys. Accordingly, berry ripening is hastened by increasing temperatures up to a maximum threshold, above which temperature becomes stressful and ripening is delayed or even prevented. Thus, supraoptimal temperatures during July–August in arid regions might slow down or even restrain carbon assimilation and sugar translocation rates (Greer and Weston, 2010; Mira de Orduña, 2010), problems that hardly occur in temperate regions. Here emerges a significant advantage of MR vineyard, where the temperature regime is relatively milder than at RN (Figure 3) and, in most of the cases, the fruit-ripening phase was shorter (Table 2 and Figure 7).

Having said that, considerable differences in the duration and in the phenological shift of the fruit-ripening phase occurred between the white and the red groups, as well as between individual cultivars within each group. White cultivars had significantly shorter fruit-ripening phase, ranging from 22 to 30 days, compared to 36–47 days in the red cultivars (Table 2). Thus, most of the white cultivars reached harvest during July, avoiding considerable portions of the mid-summer heat, with Chardonnay, Gewurztraminer, Muscat Blanc, and Semillon displaying particularly shorter ripening phases (Table 2). Yet, shorter ripening periods do not guarantee high fruit or wine quality, as the development of berry engustment may require adequate time. Harvest of the red cultivars usually occurred during August, with large differences between cultivars. While no specific red cultivars showed ripening phases adequately short to avoid the mid-summer heat, several cultivars (Petit Verdot, Malbec, Cabernet Franc, Cabernet Sauvignon, and Petit Syrah) exhibited relatively small or sometimes even opposite phenological shift, as opposed to the hyper-sensitive Tempranillo, which consistently displayed the largest shift (Figure 7). Red cultivars displaying high-temperature resilience are suitable candidates for the warmer edge of viticulture regions, as long as other conditions essential to ensure productivity and quality are satisfied. In contrast, several red cultivars such as Barbera, Dolcetto, Pinot Noir, Ruby Cabernet, Tempranillo, and Zinfandel often failed to reach the desired Brix threshold or engustment. This interruption of the ripening process, often accompanied by berry shriveling and collapse of the cluster (data not shown), was more frequent at RN, but did not occur every year among all cultivars mentioned.

Correlations between the onsets of phenological phases may be useful for the prediction of the harvest date, provided a stable phenological course. Practically, attempts to predict harvest date according to bud break onset were not successful in temperate regions (Jones and Davis, 2000; Tomasi et al., 2011). Furthermore, in the unpredictable course of winter and spring weather in arid regions, the relationships between the onsets of bud break and harvest were very poor, particularly among the red cultivars (Figure 6F). The predominant source of variation was clearly identified in the fruit-ripening phase (Figure 6M), suggesting that this is where red cultivars’ suitability to arid regions should be evaluated.

### Organic Acids and Sugars

Temperatures alter malate content in a developmental manner (Sweetman et al., 2014; Rienth et al., 2016). During pre-véraison, malate content accumulates with increasing temperature, while an inverse relation is found during ripening (Famiani et al., 2014; Sweetman et al., 2014). In the present study, contrasting results between seasons were shown for red cultivars, while white grape berry acids showed differences between locations only in 2017. These results suggest that additional factors are involved in regulating malate homeostasis in the fruit.

During ripening, major OA levels in the berry are known to decrease at a pace dependent on the genotype and the environment (Liu et al., 2006; Bigard et al., 2018). Several studies have shown a positive relationship between the loss of malate and elevated temperature (Sweetman et al., 2014; Rienth et al., 2016; Lecourieux et al., 2017). In line with what has been shown previously, the pace of OA degradation was more pronounced in warmer RN and during the hottest vintage 2017. In addition, OA in berries of the white cultivars, mostly malate, tended to be higher when the harvest date, which was determined by total soluble solid (TSS), was earlier, suggesting that the early ripening of white cultivars might better fit in hot climates. Having that said, OA concentration in berries of both white and red cultivars at harvest was predominately affected by cultivar or by the interaction of cultivar and location.

Lecourieux et al. (2017) have shown that pre-véraison heat treatment slows down sugar accumulation, due to down-regulation of sugar transporter genes that resulted in a delay of véraison onset. In the present study, this phenomenon of delayed véraison onset was absent in almost all cultivars, white cultivars in particular, most of which escape the extremely high temperatures of July–August. Still, pulp sugar composition at véraison was subject to significant varietal influences. The differences between sites in hexoses at véraison were seasonal dependent, whereas sucrose was higher in content at cooler MR site at véraison (in 2017 and 2019) and harvest (in 2018). Sucrose, in particular, displayed strong interactions between cultivar and location, confirming the significant plasticity harbored in *V. vinifera* concerning sugar metabolism toward véraison (Ollat et al., 2018). In addition, the overall, pulp sugars at harvest tended to be lower in 2018 (a year with warmer spring season) in both red and white cultivars, compared to the two other years. It is conceivable that sugar transport/accumulation is modulated already early in the season as it was shown in Merlot and Cabernet Sauvignon vines by Herrera et al. (2017); Lecourieux et al. (2017). In a different study, it was shown that early drought led to an increase in anthocyanin accumulation during ripening (Castellarin et al., 2007). We can hypothesize that early heatwaves could impose enhanced sugar catabolism in the berries toward the secondary compounds and anticipate ripening.

Hexose/sucrose ratio (HSR) is an indicator of the conversion and use of the translocated sugar, sucrose, for the metabolism of developing organs. HSR is particularly useful evaluating the performance of hexose-accumulating organs, such as the pulp of a ripening grape berry. Contrasting results are found in the literature in respect to sugar accumulation and temperature in Tempranillo berries. For instance, under controlled environment, heat treatment (28°C/18°C day/night) hastened sugar accumulation rate and significantly shortened the ripening length (Martínez-Lüscher et al., 2016b). In other studies, heat was reported to slow berry ripening in Semillon (Greer and Weedon, 2013) and in Muscat Hamburg (Carbonell-Bejerano et al., 2013). However, most of the studies investigating temperature effects on berry ripening focused on a single or very few cultivars, which may explain the unequivocal results. Furthermore, the temperature ranges studied there were much lower than those characterizing the exceptional viticulture regions explored in the present study. Our results suggest that the enzymatic apparatus responsible for sugars metabolism in the ripening fruit is highly sensitive to the temperature regime, and moreover, it may substantially differ from one cultivar to another (Basson et al., 2010; Božović et al., 2019). The higher HSR at the warmer site suggests that the stage of sucrose conversion to hexoses is thermophylic in most cultivars, but not in all. In contrast, the extension of the ripening phase and the consequent delay of harvest in many red cultivars may suggest that the foliar photosynthetic activity is damaged or inhibited under high temperature regime (Haldimann and Feller, 2004). Additionally, sucrose translocation may be significantly slowed down under high temperatures (Julius et al., 2017). The extension of the ripening phase may, in turn, lead to prolonged exposure of the berries to potentially harmful heat stress and, eventually, to berry shriveling and cluster collapse, the severity of which depends on many other berry traits (e.g., skin properties, water relations, etc.).

## Conclusion

The present study offers a unique large-scale varietal perspective of the consequences that an apparently small difference in the seasonal mean daily temperature, about 1.5°C, may induce on wine grapevine performance and berry primary metabolism. Considerable topographic gaps over small geographic distances may bring about significantly different temperature regimes on a calendric scale, eventually creating distinct terroirs within a superficially homogeneous viticulture climate region. Despite earlier onset of phenological events, and in contrast to accelerated vegetative development, berry ripening was significantly slower at warmer RN. In sensitive varieties, berries’ Brix failed to increase adequately, probably due to slower sucrose influx. In addition, the organic acids were rapidly degraded and HSR increased. Subsequently, harvest was delayed and was accompanied by low fruit quality indices.

Beyond the clear common responses to high temperature of grapevines and berry development that emerge from the present study, significant differences have occurred between the white and red groups of cultivars, as well as among cultivars within each group. Earliness seems an advantage for the white cultivars, with a much shorter ripening phase and hence avoidance of the warmest part of the season. The warmer site conditions have challenged most of the red cultivars, some of which even failed to reach adequate quality standards of ripening. Others, in contrast, exhibited impressive resilience to high temperature. Beyond cultural practices, such as shading nets and modified trellising, a careful selection of cultivars, well adapted to warm conditions, should be the utmost tool of the wine industry to meet the global climate challenge. Further research is required, however, to unravel the particular traits that make a cultivar suitable to warm conditions.

## Data Availability Statement

The raw data supporting the conclusions of this article will be made available by the authors, without undue reservation.

## Author Contributions

EH, AB, AF, and ED conceived and planned the study. KG, NS, and AB collected the berry samples in the field. AB collected the meteorological data. KG analyzed the meteorological data, prepared the berry samples for extraction, performed the sample extraction and data analysis and analysis using the GC-MS device, and wrote the body of the manuscript with AF and AB. All authors reviewed and approved the manuscript.

## Conflict of Interest

AB was employed by Ramat Negev Desert Agro-Desert Research Center, a public research station, which operates under the financial umbrella of the company Ramat Negev Works Ltd., which belongs to the public entity Ramat Negev Regional Authority. The remaining authors declare that the research was conducted in the absence of any commercial or financial relationships that could be construed as a potential conflict of interest.

## Abbreviations

BB: bud break
FS: fruit set
Vér: véraison
Har: harvest
OA: organic acids
HSR: hexoses-to-sucrose ratio
MR: Ramon
RN: Ramat Negev.
