# Supplementary material for: Temperature Shift Between Vineyards Modulates Berry Phenology and Primary Metabolism in a Varietal Collection of Wine Grapevine
Source: Front Plant Sci. 2020 Dec 17;11:588739. doi: 10.3389/fpls.2020.588739 (PMC7774500; doi:10.3389/fpls.2020.588739)
Supplement: Supplementary file 1 [file Presentation_1.PPTX]

## Slide 1
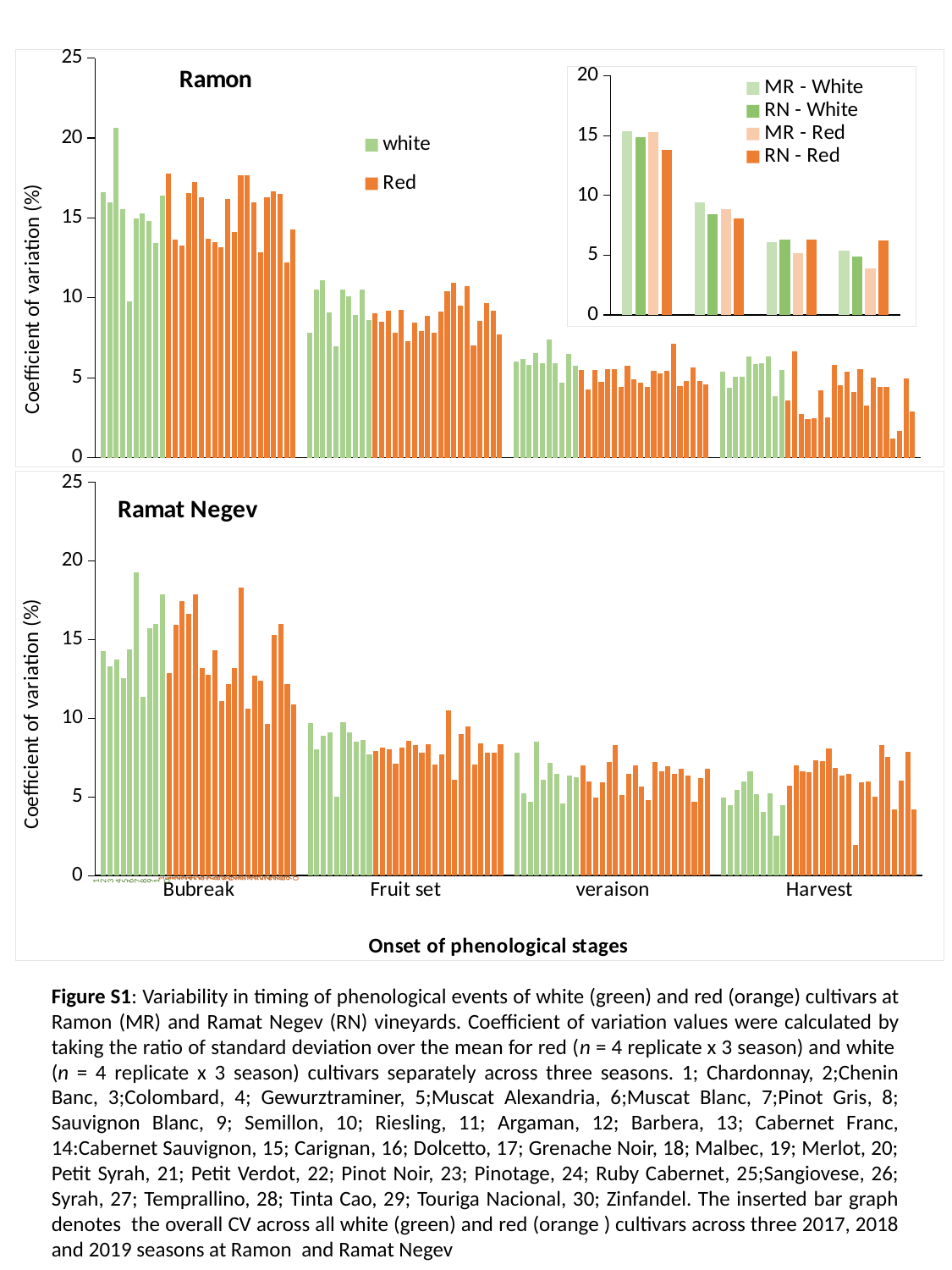

### Chart: Ramon
| Category | white | white | white | white | white | white | white | white | white | white | Red | Red | Red | Red | Red | Red | Red | Red | Red | Red | Red | Red | Red | Red | Red | Red | Red | Red | Red | Red |
|---|---|---|---|---|---|---|---|---|---|---|---|---|---|---|---|---|---|---|---|---|---|---|---|---|---|---|---|---|---|---|
| CV(Budbreak, M) | 16.604934538 | 15.955229934 | 20.646687732 | 15.547149597 | 9.7933339514 | 14.976019307 | 15.272070966 | 14.802059494 | 13.452821806 | 16.392486709 | 17.774483088 | 13.627340144 | 13.275436999 | 16.532311043 | 17.229030299 | 16.271130492 | 13.684004682 | 13.481147414 | 13.145016117 | 16.166824556 | 14.121622602 | 17.689538887 | 17.645425728 | 15.955229934 | 12.830411825 | 16.272423893 | 16.670264162 | 16.518540929 | 12.220414384 | 14.26977811 |
| CV(Fruit set, M) | 7.8205569757 | 10.530126074 | 11.10259495 | 9.1138052624 | 6.9481012612 | 10.524971211 | 10.086257065 | 8.9423142254 | 10.501293005 | 8.5977798587 | 9.02991562 | 8.4974661436 | 9.1926774785 | 7.8192525844 | 9.2289562912 | 7.3032757666 | 8.4232870542 | 7.9017441632 | 8.848252154 | 7.7942639715 | 9.1321470807 | 10.399928277 | 10.952243814 | 9.5357595499 | 10.73543645 | 7.0367723831 | 8.5542284968 | 9.6807016969 | 9.1977896785 | 7.6918761677 |
| CV( vreaison, M) | 6.0161715067 | 6.1517224915 | 5.783382783 | 6.5269162292 | 5.8963255804 | 7.3811076537 | 5.920865173 | 4.6845234937 | 6.5059096432 | 5.7757504503 | 5.483231805 | 4.2658096742 | 5.4668957428 | 4.7406992172 | 5.5563137799 | 5.519375888 | 4.4178503572 | 5.7319429549 | 4.9055860326 | 4.6869295846 | 4.4193141742 | 5.4373844594 | 5.2844017558 | 5.4472871385 | 7.1017183392 | 4.4566459696 | 4.8055211163 | 5.6717812901 | 4.7836032597 | 4.5702278457 |
| CV(Harvest, M) | 5.3929869643 | 4.3885906797 | 5.0647183907 | 5.0405975104 | 6.3151684936 | 5.8400795907 | 5.9110761247 | 6.3202467339 | 3.8679322893 | 5.4736526429 | 3.586793254 | 6.6602563142 | 2.7221333047 | 2.4057404617 | 2.4488155707 | 4.2162509272 | 2.5431912714 | 5.8172105195 | 4.5371064164 | 5.4006034959 | 4.1197040178 | 5.5587784239 | 3.2630196004 | 4.9907149409 | 4.4315913447 | 4.4163391527 | 1.2012648392 | 1.6700799883 | 4.9472465091 | 2.8728422013 |
### Chart
| Category | MR - | RN - | MR - | RN - |
|---|---|---|---|---|
| Budbreak | 15.344279403440002 | 14.839225665999999 | 15.269018764399997 | 13.774399669265 |
| Fruit set | 9.41677998884 | 8.424877820179999 | 8.84779874109 | 8.0822307322 |
| veraison | 6.064267500470001 | 6.31208288406 | 5.137626019245 | 6.33095499883 |
| Harvest | 5.36150494202 | 4.88713860829 | 3.8904841277 | 6.25994933912 |
### Chart: Ramat Negev
| Category | white | white | white | white | white | white | white | white | white | white | Red | Red | Red | Red | Red | Red | Red | Red | Red | Red | Red | Red | Red | Red | Red | Red | Red | Red | Red | Red |
|---|---|---|---|---|---|---|---|---|---|---|---|---|---|---|---|---|---|---|---|---|---|---|---|---|---|---|---|---|---|---|
| Bubreak | 14.267845968 | 13.288173734 | 13.711048728 | 12.537073448 | 14.366382066 | 19.259396016 | 11.365509708 | 15.719317683 | 15.974235149 | 17.90327416 | 12.897327096 | 15.925518077 | 17.427301985 | 16.618203404 | 17.883092699 | 13.186813187 | 12.779837526 | 14.331013481 | 11.109430554 | 12.147518868 | 13.221627304 | 18.295783753 | 10.585472304 | 12.683857377 | 12.404477911 | 9.6522007673 | 15.269143861 | 16.001828885 | 12.173132055 | 10.894412291 |
| Fruit set | 9.6692601542 | 8.0363454265 | 8.8663364307 | 9.0922151515 | 4.9885990119 | 9.7280269505 | 9.0770862614 | 8.4963248301 | 8.6178449238 | 7.6767390612 | 7.9278106321 | 8.137260095 | 8.0109127561 | 7.1017793479 | 8.1063124829 | 8.5711284219 | 8.2996426837 | 7.8217010209 | 8.3725920961 | 7.0655314589 | 7.7171711775 | 10.515762023 | 6.0614142513000004 | 9.0200109661 | 9.4797224151 | 7.0440300246 | 8.3998730948 | 7.8100699678 | 7.826925218 | 8.3549645103 |
| veraison | 7.7941539731 | 5.2230176639 | 4.7111689783 | 8.5261051904 | 6.0699729901 | 7.17208369 | 6.4531389489 | 4.5948749235 | 6.3312192837 | 6.2450931987 | 7.0235285074 | 6.0066358854 | 4.9576712184 | 5.9438011011 | 7.2309461251 | 8.2840049114 | 5.1404931071 | 6.4560277416 | 7.0247214731 | 5.6498000742 | 4.7893173928 | 7.2393583326 | 6.6464625578 | 6.922471994 | 6.4484666492 | 6.7860384536 | 6.3646401897 | 4.6799206287 | 6.2108699354 | 6.813923698 |
| Harvest | 4.9742621232 | 4.4899022749 | 5.4234121239 | 5.9731377435 | 6.601862777 | 5.1881189393 | 4.0301719425 | 5.2158253386 | 2.5177807221 | 4.4569120979 | 5.7004382189 | 7.0106294239 | 6.6413601227 | 6.5571142016 | 7.3107926544 | 7.2896181912 | 8.0529071608 | 6.8563000165 | 6.3805964781 | 6.4706409042 | 1.9666145553 | 5.9290511339 | 5.9676770257 | 4.9838938428 | 8.2708196712 | 7.5452589457 | 4.2023269447 | 6.0289283114 | 7.8381186679 | 4.1959003115 |1
2
3
4
5
6
7
8
9
10
Figure S1: Variability in timing of phenological events of white (green) and red (orange) cultivars at Ramon (MR) and Ramat Negev (RN) vineyards. Coefficient of variation values were calculated by taking the ratio of standard deviation over the mean for red (n = 4 replicate x 3 season) and white (n = 4 replicate x 3 season) cultivars separately across three seasons. 1; Chardonnay, 2;Chenin Banc, 3;Colombard, 4; Gewurztraminer, 5;Muscat Alexandria, 6;Muscat Blanc, 7;Pinot Gris, 8; Sauvignon Blanc, 9; Semillon, 10; Riesling, 11; Argaman, 12; Barbera, 13; Cabernet Franc, 14:Cabernet Sauvignon, 15; Carignan, 16; Dolcetto, 17; Grenache Noir, 18; Malbec, 19; Merlot, 20; Petit Syrah, 21; Petit Verdot, 22; Pinot Noir, 23; Pinotage, 24; Ruby Cabernet, 25;Sangiovese, 26; Syrah, 27; Temprallino, 28; Tinta Cao, 29; Touriga Nacional, 30; Zinfandel. The inserted bar graph denotes the overall CV across all white (green) and red (orange ) cultivars across three 2017, 2018 and 2019 seasons at Ramon and Ramat Negev

## Slide 2
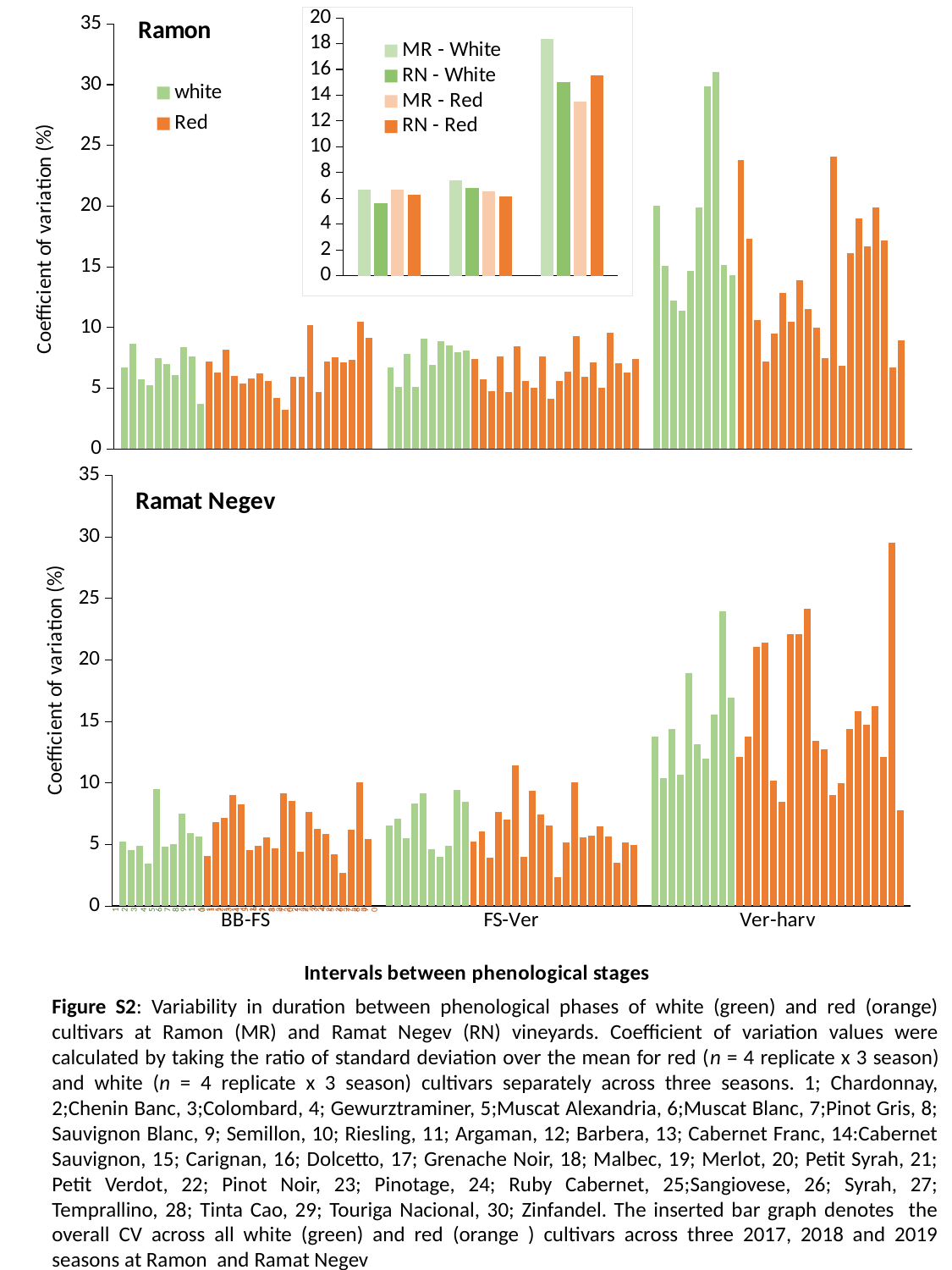

### Chart: Ramon
| Category | white | white | white | white | white | white | white | white | white | white | Red | Red | Red | Red | Red | Red | Red | Red | Red | Red | Red | Red | Red | Red | Red | Red | Red | Red | Red | Red |
|---|---|---|---|---|---|---|---|---|---|---|---|---|---|---|---|---|---|---|---|---|---|---|---|---|---|---|---|---|---|---|
| CV(BB-FS, M) | 6.7056573288 | 8.6478561993 | 5.7160371954 | 5.2568375458 | 7.4813745436 | 6.9779470911 | 6.1096801187 | 8.3495081121 | 7.6471645099 | 3.6931901721 | 7.211993725 | 6.3022206522 | 8.1724936825 | 5.9965488703 | 5.3714579301 | 5.775942372 | 6.1900505538 | 5.6274164637 | 4.2202796907 | 3.239530205 | 5.9439548787 | 5.9513970064 | 10.227753005 | 4.7049924501 | 7.2055028876 | 7.5778836874 | 7.1084468014 | 7.3105588489 | 10.458048597 | 9.1618461228 |
| CV(FS-Ver, M) | 6.6831872043 | 5.0767233574 | 7.8189298383 | 5.1027599759 | 9.0933538695 | 6.9182031963 | 8.8982052329 | 8.5034668597 | 7.9827231595 | 8.1366982234 | 7.3912369827 | 5.7148223425 | 4.7849996503 | 7.5971602058 | 4.7097501044 | 8.4754390788 | 5.5628115348 | 5.0725875421 | 7.6076951614 | 4.1090588524 | 5.5917108465 | 6.3667052812 | 9.2796543524 | 5.9220623784 | 7.115347195 | 5.0570826498 | 9.5645410667 | 7.0358900774 | 6.2814863454 | 7.3790756586 |
| CV(Ver-harv, M) | 20.041246467 | 15.043246788 | 12.242020665 | 11.357502326 | 14.674802232 | 19.90834296 | 29.87082093 | 31.051383071 | 15.132226631 | 14.32509161 | 23.817388705 | 17.281722702 | 10.584068875 | 7.2000139806 | 9.5057838072 | 12.874027405 | 10.483466706 | 13.926076147 | 11.539827279 | 9.9874824916 | 7.4779355273 | 24.067856187 | 6.8764178467 | 16.155758191 | 18.959397301 | 16.646047852 | 19.908567347 | 17.174453313 | 6.7002521017 | 8.960904412 |
### Chart
| Category | MR - | RN - | MR - | RN - |
|---|---|---|---|---|
| BB-FS | 6.658525281679999 | 5.64839856537 | 6.687915921530001 | 6.281507957114999 |
| FS-Ver | 7.421425091719999 | 6.794838114700001 | 6.53095586533 | 6.167791398140001 |
| Ver-harv | 18.364668368 | 14.980993267299999 | 13.506372408855 | 15.562738756529999 |
### Chart: Ramat Negev
| Category | white | white | white | white | white | white | white | white | white | white | Red | Red | Red | Red | Red | Red | Red | Red | Red | Red | Red | Red | Red | Red | Red | Red | Red | Red | Red | Red |
|---|---|---|---|---|---|---|---|---|---|---|---|---|---|---|---|---|---|---|---|---|---|---|---|---|---|---|---|---|---|---|
| BB-FS | 5.213303076 | 4.5491275219 | 4.8975392446 | 3.4520128764 | 9.5114241187 | 4.804999801 | 5.0153314893 | 7.4744214626 | 5.9250882076 | 5.6407378556 | 4.0369760281 | 6.841598245 | 7.1784942592 | 9.0582258807 | 8.2749747374 | 4.5454545455 | 4.8655507955 | 5.5654075262 | 4.6922801186 | 9.1322760332 | 8.5532915948 | 4.436785405 | 7.653911028 | 6.2884998097 | 5.8868708729 | 4.1718623625 | 2.6889352274 | 6.2002979462 | 10.080241284 | 5.4782254424 |
| FS-Ver | 6.5445801483 | 7.0718631804 | 5.4847395618 | 8.2996426837 | 9.167909512 | 4.5987085493 | 3.9981468357 | 4.8689901863 | 9.409547085 | 8.5042534045 | 5.2442444601 | 6.0840391195 | 3.9256925525 | 7.641852558 | 7.034682177 | 11.419683489 | 4.0182767368 | 9.3319304935 | 7.4206097215 | 6.5405405852 | 2.3364014211 | 5.2016339497 | 10.051540542 | 5.5718335316 | 5.7400192865 | 6.5006567916 | 5.668939272 | 3.4943836192 | 5.1518278859 | 4.9770397701 |
| Ver-harv | 13.749033305 | 10.400215581 | 14.37169741 | 10.657815671 | 18.971500908 | 13.185161857 | 12.017982409 | 15.552010061 | 23.974065627 | 16.930449844 | 12.110549342 | 13.767449087 | 21.08731066 | 21.414705677 | 10.174792381 | 8.4449421906 | 22.116726883 | 22.140532878 | 24.139725076 | 13.400504203 | 12.724079221 | 9.0436013504 | 10.000497344 | 14.364413216 | 15.811548402 | 14.766937993 | 16.281761141 | 12.15460662 | 29.54326915 | 7.7668223156 |16
17
18
19
20
24
22
23
25
21
11
12
13
14
15
26
27
28
29
30
1
2
3
4
5
6
7
8
9
10
Figure S2: Variability in duration between phenological phases of white (green) and red (orange) cultivars at Ramon (MR) and Ramat Negev (RN) vineyards. Coefficient of variation values were calculated by taking the ratio of standard deviation over the mean for red (n = 4 replicate x 3 season) and white (n = 4 replicate x 3 season) cultivars separately across three seasons. 1; Chardonnay, 2;Chenin Banc, 3;Colombard, 4; Gewurztraminer, 5;Muscat Alexandria, 6;Muscat Blanc, 7;Pinot Gris, 8; Sauvignon Blanc, 9; Semillon, 10; Riesling, 11; Argaman, 12; Barbera, 13; Cabernet Franc, 14:Cabernet Sauvignon, 15; Carignan, 16; Dolcetto, 17; Grenache Noir, 18; Malbec, 19; Merlot, 20; Petit Syrah, 21; Petit Verdot, 22; Pinot Noir, 23; Pinotage, 24; Ruby Cabernet, 25;Sangiovese, 26; Syrah, 27; Temprallino, 28; Tinta Cao, 29; Touriga Nacional, 30; Zinfandel. The inserted bar graph denotes the overall CV across all white (green) and red (orange ) cultivars across three 2017, 2018 and 2019 seasons at Ramon and Ramat Negev
