# Supplementary material for: Temperature Shift Between Vineyards Modulates Berry Phenology and Primary Metabolism in a Varietal Collection of Wine Grapevine
Source: Front Plant Sci. 2020 Dec 17;11:588739. doi: 10.3389/fpls.2020.588739 (PMC7774500; doi:10.3389/fpls.2020.588739)
Supplement: Supplementary file 2 [file Table_1.DOCX]

Supplementary Table 1: The onset of major phenological events in red and white cultivars at Ramon (MR) and Ramat Negev (RN) from 2017-2019 growing seasons.

| Cultivar | | Bud break | | | | | Fruit set | | | | | |
| --- | --- | --- | --- | --- | --- | --- | --- | --- | --- | --- | --- | --- |
|  |  | 2017 | | | 2018 | | 2017 | | 2018 | | 2019 | |
|  |  | MR | RN | | MR | RN | MR | RN | MR | RN | MR | RN |
| Red | Arg | 01-Apr±1.7 | 22-Mar±0.0 | | 16-Mar±0.6 | 11-Mar±0.3 | 25-May±1.8 | 09-May±0.6 | 05-May±1.5 | 28-Apr±1.2 | 22-May±0.3 | 13-May±0.0 |
|  | Bar | 30-Mar±1.2 | 22-Mar±0.0 | | 19-Mar±1.4 | 09-Mar±1.0 | 17-May±1.0 | 02-May±0.6 | 02-May±1.7 | 24-Apr±0.3 | 19-May±0.7 | 12-May±0.9 |
|  | CF | 27-Mar±0.0 | 21-Mar±1.0 | | 16-Mar±0.8 | 08-Mar±0.5 | 21-May±0.6 | 03-May±0.0 | 03-May±1.5 | 25-Apr±0.0 | 21-May±0.0 | 12-May±0.8 |
|  | CS | 03-Apr±0.6 | 26-Mar±1.5 | | 19-Mar±1.4 | 12-Mar±0.3 | 24-May±1.3 | 09-May±0.5 | 07-May±0.0 | 30-Apr±1.7 | 22-May±0.3 | 16-May±0.5 |
|  | Car | 29-Mar±1.0 | 21-Mar±0.9 | | 14-Mar±0.8 | 07-Mar±0.3 | 22-May±1.0 | 02-May±0.6 | 03-May±1.4 | 25-Apr±0.0 | 22-May±0.3 | 12-May±0.8 |
|  | Dol | 01-Apr±2.0 | 23-Mar±0.6 | | 18-Mar±1.5 | 12-Mar±0.7 | 21-May±1.4 | 06-May±1.7 | 06-May±1.5 | 25-Apr±0.0 | 20-May±0.7 | 13-May±0.3 |
|  | GN | 25-Mar±0.8 | 19-Mar±0.8 | | 14-Mar±0.7 | 09-Mar±1.4 | 19-May±1.4 | 03-May±0.0 | 03-May±1.8 | 25-Apr±0.0 | 20-May±0.8 | 13-May±0.0 |
|  | Mal | 29-Mar±1.3 | 22-Mar±0.0 | | 18-Mar±0.9 | 11-Mar±1.2 | 18-May±1.8 | 03-May±0.0 | 04-May±1.2 | 25-Apr±0.0 | 21-May±0.0 | 12-May±1.5 |
|  | Mer | 27-Mar±0.0 | 22-Mar±0.0 | | 16-Mar±0.3 | 13-Mar±0.8 | 16-May±0.0 | 05-May±1.3 | 01-May±0.0 | 25-Apr±0.0 | 21-May±0.0 | 13-May±0.0 |
|  | PS | 31-Mar±0.3 | 22-Mar±0.3 | | 17-Mar±0.6 | 12-Mar±0.9 | 20-May±0.4 | 04-May±1.3 | 05-May±1.4 | 29-Apr±1.5 | 21-May±0.0 | 13-May±0.3 |
|  | PV | 28-Mar±1.0 | 25-Mar±0.5 | | 16-Mar±0.7 | 13-Mar±1.0 | 19-May±1.8 | 03-May±0.0 | 01-May±0.0 | 27-Apr±2.0 | 20-May±0.8 | 13-May±0.0 |
|  | PN | 29-Mar±1.3 | 21-Mar±0.6 | | 14-Mar±0.3 | 07-Mar±0.3 | 13-May±1.7 | 01-May±0.8 | 25-Apr±0.3 | 18-Apr±0.0 | 16-May±0.5 | 9-May±1.4 |
|  | Pt | 01-Apr±0.5 | 23-Mar±0.5 | | 16-Mar±1.7 | 14-Mar±0.9 | 27-May±1.5 | 08-May±2.3 | 03-May±1.5 | 01-May±0.6 | 22-May±0.4 | 15-May±0.5 |
|  | RC | 27-Mar±0.0 | 20-Mar±1.7 | | 14-Mar±0.5 | 11-Mar±1.5 | 11-May±1.2 | 01-May±1.2 | 26-Apr±0.5 | 23-Apr±1.9 | 16-May±0.3 | 11-May±1.4 |
|  | Sg | 22-Mar±0.6 | 18-Mar±1.3 | | 12-Mar±0.6 | 10-Mar±2.2 | 14-May±2.0 | 01-May±0.6 | 26-Apr±1.3 | 22-Apr±2.1 | 20-May±0.5 | 11-May±0.8 |
|  | Syrah | 01-Apr±1.9 | 22-Mar±0.6 | | 18-Mar±1.9 | 14-Mar±0.8 | 22-May±0.6 | 06-May±1.4 | 07-May±0.0 | 30-Apr±1.5 | 21-May±0.0 | 14-May±0.6 |
|  | Temp | 05-Apr±0.8 | 28-Mar±0.5 | | 20-Mar±1.6 | 14-Mar±0.6 | 29-May±1.7 | 08-May±0.0 | 08-May±0.3 | 25-Apr±0.0 | 22-May±0.3 | 13-May±0.0 |
|  | TC | 26-Mar±1.0 | 18-Mar±1.5 | | 13-Mar±0.5 | 07-Mar±0.3 | 14-May±1.8 | 02-May±0.5 | 29-Apr±1.4 | 24-Apr±0.3 | 21-May±0.5 | 11-May±0.8 |
|  | TN | 02-Apr±0.9 | 24-Mar±2.0 | | 21-Mar±0.3 | 14-Mar±0.3 | 23-May±2.1 | 02-May±0.7 | 03-May±0.9 | 27-Apr±2.0 | 20-May±0.5 | 13-May±0.0 |
|  | Zin | 01-Apr±1.1 | 21-Mar±2.1 | | 20-Mar±1.8 | 14-Mar±0.6 | 20-May±4.0 | 03-May±0.5 | 06-May±1.0 | 25-Apr±0.0 | 19-May±0.3 | 13-May±0.0 |
|  | Average | 31-Mar±0.4 | 22-Mar±0.3 | | 16-Mar±0.4 | 11-Mar±0.3 | 20-May±0.6 | 04-May±0.3 | 02-May±0.5 | 25-Apr±0.4 | 20-May±0.2 | 13-May±0.2 |
|  |  |  |  |  | |  |  |  |  |  |  |  |
| White | Chardonnay | 28-Mar±0.0 | 17-Mar±0.8 | 13-Mar±0.6 | | 07-Mar±0.3 | 16-May±0.0 | 01-May±0.8 | 03-May±2.5 | 18-Apr±0.3 | 19-May±0.9 | 8-May±0.8 |
|  | Chenin B | 27-Mar±0.5 | 16-Mar±0.8 | 14-Mar±0.5 | | 06-Mar±0.3 | 22-May±1.6 | 30-Apr±0.0 | 30-Apr±0.5 | 24-Apr±0.0 | 21-May±0.0 | 11-May±0.8 |
|  | Colombard | 31-Mar±1.3 | 16-Mar±0.8 | 13-Mar±0.5 | | 06-Mar±0.4 | 18-May±1.3 | 30-Apr±0.4 | 27-Apr±1.2 | 20-Apr±1.4 | 20-May±0.9 | 8-May±0.9 |
|  | Gewurzt | 27-Mar±0.5 | 16-Mar±0.8 | 14-Mar±0.5 | | 07-Mar±0.3 | 15-May±1.7 | 29-Apr±0.3 | 30-Apr±0.5 | 19-Apr±0.3 | 20-May±0.9 | 7-May±0.0 |
|  | Muscat A | 03-Apr±0.6 | 27-Mar±1.0 | 25-Mar±0.9 | | 16-Mar±2.9 | 26-May±1.7 | 10-May±0.5 | 11-May±1.9 | 04-May±0.3 | 25-May±0.3 | 15-May±0.8 |
|  | Muscat B | 27-Mar±0.0 | 21-Mar±1.0 | 14-Mar±0.6 | | 06-Mar±0.5 | 12-May±1.8 | 30-Apr±0.3 | 26-Apr±1.0 | 18-Apr±0.0 | 19-May±0.8 | 8-May±0.8 |
|  | Pinot Gris | 27-Mar±0.0 | 17-Mar±0.8 | 14-Mar±0.0 | | 09-Mar±0.3 | 11-May±1.8 | 30-Apr±0.3 | 27-Apr±1.0 | 18-Apr±0.0 | 19-May±0.9 | 6-May±0.3 |
|  | Semillon | 28-Mar±1.3 | 22-Mar±0.0 | 18-Mar±2.1 | | 09-Mar±0.0 | 19-May±1.6 | 07-May±2.0 | 29-Apr±1.2 | 25-Apr±0.3 | 21-May±0.0 | 12-May±0.8 |
|  | Sauvignon B | 29-Mar±0.6 | 25-Mar±1.3 | 17-Mar±1.9 | | 12-Mar±0.9 | 22-May±0.5 | 06-May±1.8 | 03-May±1.8 | 25-Apr±0.0 | 21-May±0.0 | 13-May±0.0 |
|  | Riesling | 02-Apr±0.3 | 25-Mar±1.3 | 17-Mar±0.8 | | 10-Mar±0.5 | 17-May±1.3 | 06-May±1.4 | 02-May±0.5 | 25-Apr±0.3 | 20-May±0.8 | 10-May±1.2 |
|  | Average | 29-Mar±0.4 | 20-Mar±0.7 | 15-Mar±0.6 | | 08-Mar±0.6 | 17-May±0.8 | 02-May±0.6 | 30-Apr±0.8 | 22-Apr±0.8 | 20-May±0.4 | 10-May±0.5 |

Table S1: Continued

| Cultivar | | véraison | | | |  | | Harvest | | | | | | |
| --- | --- | --- | --- | --- | --- | --- | --- | --- | --- | --- | --- | --- | --- | --- |
|  |  | 2017 | | 2018 | | 2019 | | 2017 | | 2018 | | 2019 | | |
|  |  | MR | RN | MR | RN | MR | RN | MR | RN | MR | RN | MR | RN |  |
| Red | Arg | 07-Jul±0.9 | 28-Jun±0.8 | 20-Jun±0.4 | 10-Jun±1.7 | 7-Jul±0.8 | 1-Jul±1.3 | 3-Aug±1.2 | 02-Aug±1.7 | 07-Aug±1.0 | 20-Jul±1.8 | 18-Aug±0.0 | 12-Aug±2.0 |  |
|  | Bar | 08-Jul±1.0 | 30-Jun±1.0 | 25-Jun±1.7 | 16-Jun±0.3 | 8-Jul±0.4 | 6-Jul±0.3 | 13-Aug±0.8 | 18-Aug±1.8 | 21-Jul±0.5 | 24-Jul±2.9 | 18-Aug±0.0 | 20-Aug±1.2 |  |
|  | CF | 13-Jul±1.3 | 01-Jul±0.8 | 25-Jun±0.6 | 20-Jun±0.0 | 11-Jul±0.4 | 7-Jul±0.6 | 17-Aug±1.5 | 18-Aug±1.8 | 05-Aug±0.6 | 20-Jul±0.8 | 15-Aug±0.5 | 10-Aug±0.0 |  |
|  | CS | 07-Jul±1.6 | 26-Jun±2.6 | 22-Jun±0.8 | 18-Jun±1.0 | 6-Jul±0.3 | 7-Jul±0.6 | 15-Aug±1.4 | 21-Aug±2.1 | 08-Aug±0.0 | 25-Jul±2.3 | 18-Aug±0.0 | 16-Aug±0.0 |  |
|  | Car | 10-Jul±0.4 | 28-Jun±0.0 | 21-Jun±0.5 | 15-Jun±1.8 | 6-Jul±0.0 | 9-Jul±0.0 | 20-Aug±0.5 | 21-Aug±2.1 | 09- Aug±0.8 | 30-Jul±0.0 | 18-Aug±0.0 | 31-Aug±0.0 |  |
|  | Dol | 02-Jul±2.0 | 17-Jun±1.1 | 18-Jun±1.7 | 08-Jun±1.0 | 5-Jul±0.3 | 4-Jul±0.0 | 3-Aug±1.2 | 03-Aug±2.0 | 23-Jul±1.8 | 24-Jul±3.1 | 10-Aug±0.0 | 23-Aug±0.0 |  |
|  | GN | 07-Jul±1.0 | 29-Jun±0.6 | 24-Jun±0.8 | 18-Jun±1.0 | 9-Jul±0.3 | 5-Jul±0.0 | 12-Aug±0.8 | 06-Aug±0.0 | 08-Aug±0.5 | 28-Jul±1.8 | 20-Aug±0.8 | 31-Aug±0.0 |  |
|  | Mal | 08-Jul±2.8 | 15-Jun±1.3 | 21-Jun±0.8 | 15-Jun±0.4 | 7-Jul±0.8 | 3-Jul±1.2 | 13-Aug±0.0 | 01-Aug±1.8 | 25-Jul±2.0 | 13-Jul±1.2 | 19-Aug±0.8 | 10-Aug±0.0 |  |
|  | Mer | 07-Jul±1.0 | 21-Jun±2.3 | 21-Jun±0.8 | 13-Jun±1.8 | 4-Jul±0.3 | 5-Jul±0.0 | 11-Aug±0.7 | 30-Jul±1.7 | 23-Jul±2.0 | 10-Jul±0.5 | 2-Aug±0.0 | 2-Aug±0.5 |  |
|  | PS | 04-Jul±0.5 | 23-Jun±2.0 | 20-Jun±0.3 | 13-Jun±0.0 | 4-Jul±0.3 | 1-Jul±1.0 | 12-Aug±0.7 | 11-Aug±2.4 | 23-Jul±1.8 | 21-Jul±1.8 | 13-Aug±1.7 | 16-Aug±0.0 |  |
|  | PV | 10-Jul±0.8 | 30-Jun±0.3 | 26-Jun±0.6 | 24-Jun±0.6 | 10-Jul±0.5 | 11-Jul±0.0 | 26-Aug±1.0 | 14-Aug±2.5 | 12-Aug±0.0 | 11-Aug±1.5 | 31-Aug±0.0 | 16-Aug±0.0 |  |
|  | PN | 29-Jun±1.0 | 15-Jun±0.8 | 13-Jun±0.0 | 05-Jun±0.0 | 28-Jun±0.6 | 28-Jun±1.1 | 26-Jul±1.3 | 15-Jul±0.8 | 25-Jul±1.8 | 11-Jul±1.2 | 14-Aug±2.3 | 2-Aug±0.0 |  |
|  | Pt | 08-Jul±0.0 | 28-Jun±0.0 | 21-Jun±1.3 | 15-Jun±1.4 | 7-Jul±0.5 | 8-Jul±0.5 | 21-Aug±1.8 | 18-Aug±1.3 | 08-Aug±0.0 | 28-Jul±1.5 | 19-Aug±0.8 | 21-Aug±1.4 |  |
|  | RC | 28-Jun±0.0 | 14-Jun±0.0 | 13-Jun±0.3 | 05-Jun±0.0 | 30-Jun±1.5 | 27-Jun±0.9 | 6-Aug±4.3 | 21-Jul±2.8 | 27-Jul±1.8 | 10-Jul±1.7 | 14-Aug±2.3 | 28-Jul±1.8 |  |
|  | Sg | 04-Jul±2.3 | 19-Jun±0.0 | 15-Jun±1.8 | 13-Jun±0.3 | 6-Jul±0.6 | 3-Jul±1.1 | 10-Aug±1.0 | 09-Aug±1.7 | 02-Aug±3.8 | 17-Jul±0.5 | 20-Aug±0.9 | 21-Aug±1.4 |  |
|  | Syrah | 06-Jul±1.2 | 27-Jun±0.5 | 23-Jun±1.1 | 13-Jun±1.9 | 7-Jul±0.5 | 5-Jul±0.3 | 2-Aug±1.2 | 14-Aug±1.8 | 27-Jul±3.6 | 22-Jul±0.8 | 13-Aug±1.7 | 23-Aug±0.0 |  |
|  | Tem | 04-Jul±0.6 | 21-Jun±1.7 | 19-Jun±0.6 | 09-Jun±1.2 | 2-Jul±1.0 | 29-Jun±0.7 | 2-Aug±1.0 | 14-Aug±6.7 | 31-Jul±1.8 | 14-Aug±0.0 | 2-Aug±0.0 | 23-Aug±0.0 |  |
|  | TC | 08-Jul±1.0 | 01-Jul±0.5 | 21-Jun±0.8 | 20-Jun±0.0 | 8-Jul±0.8 | 6-Jul±0.4 | 17-Aug±1.3 | 18-Aug±1.8 | 12-Aug±0.0 | 05-Aug±2.4 | 18-Aug±1.0 | 31-Aug±0.0 |  |
|  | TN | 07-Jul±0.9 | 19-Jun±0.0 | 22-Jun±0.8 | 16-Jun±1.0 | 7-Jul±0.6 | 5-Jul±0.6 | 19-Aug±0.5 | 27-Aug±0.0 | 30-Jul±0.0 | 24-Jul±0.7 | 17-Aug±1.0 | 15-Aug±0.7 |  |
|  | Zin | 07-Jul±1.2 | 19-Jun±0.0 | 23-Jun±1.0 | 14-Jun±0.0 | 6-Jul±0.3 | 5-Jul±0.0 | 18-Aug±1.8 | 09-Aug±1.3 | 08-Aug±0.5 | 30-Jul±0.8 | 16-Aug±2.0 | 18-Aug±0.5 |  |
|  | Average | 06-Jul±0.5 | 24-Jun±0.7 | 20-Jun±0.4 | 14-Jun±0.6 | 6-Jul±0.4 | 4-Jul±0.4 | 12-Aug±0.8 | 09-Aug±1.3 | 01-Aug±0.9 | 24-Jul±1.2 | 16Aug±0.7 | 17-Aug±1.1 |  |
|  | |  |  |  |  |  |  |  |  |  |  |  |  |  |
| White | Chardonnay | 05-Jul±0.0 | 23-Jun±2.0 | 17-Jun±1.6 | 14-Jun±0.5 | 5-Jul±0.3 | 28-Jun±0.8 | 2-Aug±0.8 | 14-Jul±4 | 12-Jul±1.7 | 01-Jul±0.3 | 23-Jul±0.9 | 19-Jul±0.3 |  |
|  | Chenin B | 10-Jul±1.8 | 26-Jun±0.3 | 20-Jun±0.3 | 05-Jun±0.0 | 6-Jul±0.3 | 30-Jun±0.3 | 6-Aug±0.0 | 30-Jul | 23-Jul±0.0 | 16-Jul±0.8 | 10-Aug±0.0 | 31-Jul±3.0 |  |
|  | Colombard | 07-Jul±2.3 | 21-Jun±1.8 | 20-Jun±1.7 | 13-Jun±0.5 | 4-Jul±0.0 | 27-Jun±0.5 | 6-Aug±0.0 | 04-Aug±4 | 16-Jul±1.8 | 15-Jul±0.0 | 29-Jul±1.3 | 2-Aug±0.0 |  |
|  | Gewurzt | 30-Jun±1.7 | 17-Jun±0.9 | 14-Jun±0.0 | 14-Jun±0.0 | 4-Jul±0.3 | 25-Jun±0.6 | 26-Jul±2.8 | 09-Jul±1 | 09-Jul±0.0 | 27-Jul±1.0 | 25-Jul±0.0 | 19-Jul±0.0 |  |
|  | Muscat A | 17-Jul±0.5 | 05-Jul±0.3 | 26-Jun±0.3 | 30-Jun±1.9 | 10-Jul±1.7 | 8-Jul±0.9 | 15-Aug±0.0 | 17-Aug±4 | 20-Jul±1.8 | 20-Jul±1.2 | 10-Aug±0.5 | 9-Aug±0.9 |  |
|  | Muscat B | 05-Jul±0.0 | 20-Jun±1.1 | 13-Jun±0.0 | 18-Jun±1.0 | 5-Jul±0.5 | 27-Jun±0.3 | 1-Aug±1.2 | 12-Jul±1 | 09-Jul±0.0 | 01-Jul±0.0 | 23-Jul±1.0 | 21-Jul±1.8 |  |
|  | Pinot G | 06-Jul±0.9 | 17-Jun±1.0 | 18-Jun±1.7 | 05-Jun±0.5 | 5-Jul±0.4 | 27-Jun±0.4 | 23-Jul±0.0 | 11-Jul±3 | 18-Jul±1.8 | 05-Jul±1.0 | 10-Aug±0.0 | 19-Jul±0.0 |  |
|  | Semillon | 13-Jul±1.0 | 03-Jul±0.3 | 21-Jun±0.9 | 05-Jun±0.0 | 7-Jul±0.3 | 30-Jun±0.3 | 31-Jul±0.5 | 18-Jul±1 | 15-Jul±0.0 | 08-Jul±0.0 | 25-Jul±0.3 | 26-Jul±0.5 |  |
|  | Sauvignon | 05-Jul±0.0 | 24-Jun±0.7 | 20-Jun±0.0 | 13-Jun±0.0 | 3-Jul±0.3 | 28-Jun±0.3 | 23-Jul±0.0 | 15-Jul±1 | 15-Jul±0.0 | 07-Jul±1.3 | 10-Aug±0.5 | 27-Jul±0.8 |  |
|  | Riesling | 10-Jul±1.7 | 03-Jul±1.0 | 20-Jun±0.8 | 13-Jun±0.0 | 6-Jul±0.0 | 29-Jun±0.3 | 6-Aug±0.0 | 27-Jul±5 | 19-Jul±1.5 | 16-Jul±0.6 | 10-Aug±0.5 | 3-Aug±0.5 |  |
|  | Average | 7-Jul±4.1 | 24-Jun±1.1 | 18-Jun±0.6 | 12-Jun±0.9 | 5-Jul±0.3 | 29-Jun±0.5 | 4-Aug±1.1 | 21-Jul±2.0 | 15-Jul±0.8 | 11-Jul±1.2 | 2-Aug±1.3 | 27-Jul±1.2 |  |

Data are means ±SE of four replicates

Table S2: Correlations (R^2^) values between the onset of phenological events in red and white cultivars grown at Ramon and Ramat Negev vineyards over the period from 2017-2019.

| Cultivar |  | BB with Fs | BB with Ver | BB with Harvest | Fs with Ver | Fs with Harvest | Ver with Harvest |
| --- | --- | --- | --- | --- | --- | --- | --- |
| Red | Arg | 0.92 | 0.91 | 0.23 | 0.86 | 0.34 | 0.54 |
|  | Bar | 0.89 | 0.92 | 0.46 | 0.84 | 0.44 | 0.60 |
|  | CF | 0.83 | 0.88 | 0.84 | 0.92 | 0.66 | 0.60 |
|  | CS | 0.81 | 0.91 | 0.57 | 0.79 | 0.60 | 0.56 |
|  | Car | 0.83 | 0.95 | 0.77 | 0.87 | 0.32 | 0.72 |
|  | Dol | 0.88 | 0.87 | 0.46 | 0.82 | 0.30 | 0.56 |
|  | GN | 0.86 | 0.95 | 0.56 | 0.90 | 0.47 | 0.54 |
|  | Mal | 0.86 | 0.62 | 0.94 | 0.88 | 0.88 | 0.71 |
|  | Mer | 0.93 | 0.80 | 0.89 | 0.85 | 0.72 | 0.73 |
|  | PS | 0.82 | 0.91 | 0.73 | 0.89 | 0.51 | 0.67 |
|  | PV | 0.75 | 0.85 | 0.60 | 0.88 | 0.78 | 0.56 |
|  | PN | 0.95 | 0.92 | 0.32 | 0.94 | 0.58 | 0.62 |
|  | Pt | 0.88 | 0.90 | 0.71 | 0.75 | 0.52 | 0.82 |
|  | RC | 0.91 | 0.83 | 0.45 | 0.90 | 0.60 | 0.66 |
|  | Sg | 0.75 | 0.65 | 0.50 | 0.90 | 0.67 | 0.67 |
|  | Syrah | 0.82 | 0.82 | 0.18 | 0.79 | 0.19 | 0.52 |
|  | Tem | 0.86 | 0.87 | 0.11 | 0.86 | 0.15 | 0.04 |
|  | TC | 0.84 | 0.86 | 0.50 | 0.82 | 0.30 | 0.47 |
|  | TN | 0.83 | 0.84 | 0.51 | 0.94 | 0.39 | 0.26 |
|  | Zin | 0.83 | 0.82 | 0.73 | 0.90 | 0.66 | 0.82 |
|  |  |  |  |  |  |  |  |
| White | Chardonnay | 0.86 | 0.94 | 0.80 | 0.89 | 0.68 | 0.70 |
|  | Chenin B | 0.92 | 0.95 | 0.91 | 0.90 | 0.79 | 0.84 |
|  | Colombard | 0.95 | 0.90 | 0.62 | 0.92 | 0.47 | 0.50 |
|  | Gewurzt | 0.94 | 0.94 | 0.92 | 0.94 | 0.93 | 0.96 |
|  | Muscat A | 0.76 | 0.88 | 0.54 | 0.73 | 0.34 | 0.70 |
|  | Muscat B | 0.91 | 0.93 | 0. 64 | 0.94 | 0.66 | 0.70 |
|  | Pinot Gris | 0.94 | 0.94 | 0.41 | 0.89 | 0.67 | 0.59 |
|  | Sauvignon B | 0.79 | 0.83 | 0.13 | 0.96 | 0.42 | 0.36 |
|  | Semillon | 0.83 | 0.85 | 0.01 | 0.82 | 0.16 | 0.07 |
|  | Riesling | 0.94 | 0.94 | 0.86 | 0.79 | 0.86 | 0.78 |

BB, Budbreak; FS, fruit set; Ver, véraison; Har, Harvest. The correlation analysis was done using raw data of four replicates (each consisting of 8-9 plants) at Ramon and Ramat Negev vineyards in 2017, 2018, and 2019 seasons.

Table S3: A factorial analysis of variance for the onset of phenological phases and their duration in white cultivars over the period from 2017- 2019 growing seasons

|  | Source | Intervals between phenological stages | | | Onset of phenological events | | | |
| --- | --- | --- | --- | --- | --- | --- | --- | --- |
|  |  | BB-FS | FS-Ver | Ver-Har | BB | FS | Ver | Har |
| Means of main factors | Chardonnay | 46.9±1.13 | 48.2±0.68 | 21.7±0.78 | 15-Mar±2.01 | 5-May±2.24 | 23-Jun±2.32 | 16-Jul±2.17 |
|  | Chenin B | 49.1±1.00 | 50.5±0.74 | 32.2±0.84 | 15-Mar±1.99 | 7-May±2.31 | 27-Jun±1.97 | 29-Jul±1.86 |
|  | Colombard | 46.3±0.69 | 50.7±0.77 | 32.3±1.38 | 15-Mar±2.45 | 5-May±2.40 | 25-Jun±1.90 | 27-Jul±1.89 |
|  | Gewurzt | 45.6±0.72 | 45.7±0.65 | 24.0±0.54 | 15-Mar±1.95 | 4-May±2.24 | 13-Jun±6.43 | 14-Jul±2.22 |
|  | Muscat A | 47.8±1.08 | 49.8±1.02 | 32.5±1.39 | 25-Mar±1.89 | 14-May±1.79 | 3-Jul±2.02 | 4-Aug±2.42 |
|  | Muscat B | 42.9±0.77 | 49.5±0.58 | 22.9±0.80 | 16-Mar±2.11 | 3-May±2.26 | 22-Jun±2.41 | 16-Jul±2.19 |
|  | Pinot G | 43.0±0.65 | 50.7±0.73 | 26.3±1.29 | 16-Mar±1.83 | 3-May±2.18 | 22-Jun±2.25 | 19-Jul±2.46 |
|  | Sauvignon B | 46.8±1.23 | 47.0±0.67 | 26.2±1.31 | 20-Mar±1.91 | 9-May±2.09 | 25-Jun±1.63 | 20-Jul±2.34 |
|  | Semillon | 46.7±0.79 | 51.5±0.90 | 20.9±0.89 | 19-Mar±1.97 | 8-May±2.16 | 29-Jun±2.12 | 20-Jul±1.61 |
|  | Riesling | 44.8±0.56 | 51.2±0.90 | 30.1±0.95 | 20-Mar±2.30 | 8-May±1.96 | 28-Jun±1.96 | 28-Jul±1.98 |
|  | MR | 47.8±0.44 | 48.5±0.37 | 25.7±0.57 | 22-Mar±0.88 | 12-May±0.92 | 29-Jun±1.55 | 26-Jul±0.98 |
|  | RN | 44.2±0.34 | 50.4±0.37 | 28.1±0.36 | 13-Mar±0.78 | 1-May±0.78 | 20-Jun±0.92 | 18-Jul±1.13 |
|  | 2017 | 46.5±0.50 | 51.6±0.52 | 25.1±0.93 | 24-Mar±0.68 | 9-May±1.00 | 28-Jun±2.17 | 26-Jul±1.27 |
|  | 2018 | 45.5±0.37 | 48.8±0.42 | 27.8±0.42 | 11-Mar±0.57 | 25-Apr±0.71 | 13-Jun±0.75 | 11-Jul±0.83 |
|  | 2019 | - | 48.0±0.33 | 27.7±0.77 | - | 15-May±0.67 | 2-Jul±0.48 | 30-Jul±0.93 |
| ANOVA P of main factors and their interactions | Cultivar | <.0001 | <.0001 | <.0001 | 0.0275 | <.0001 | <.0001 | <.0001 |
|  | Location | <.0001 | <.0001 | <.0001 | <.0001 | <.0001 | <.0001 | <.0001 |
|  | Year | 0.0159 | <.0001 | <.0001 | <.0001 | <.0001 | <.0001 | <.0001 |
|  | Location*year | <.0001 | <.0001 | <.0001 | ns | <.0001 | <.0001 | <.0001 |
|  | Cultivar*Location | 0.0293 | 0.0004 | <.0001 | ns | 0.0004 | <.0001 | <.0001 |
|  | Cultivar* Year | ns | <.0001 | <.0001 | ns | <.0001 | <.0001 | <.0001 |
|  | Cultivar*Location*year | 0.0005 | 0.0050 | <.0001 | ns | 0.0002 | <.0001 | <.0001 |

Values are elapsed days mean ±SE, (*n* = 4 replicates x 2 location x 3 seasons). BB, Budbreak; FS, fruit set; Ver, véraison; Har, Harvest; RN, Ramat Negev; MR, Ramon. ns, not significant.

Table S4: A factorial analysis of variance for the onset of phenological phases and their duration in red cultivars over the period from 2017- 2019 growing seasons.

|  | Source | Intervals between phenology | | | On set of phenological events | | |  |
| --- | --- | --- | --- | --- | --- | --- | --- | --- |
|  |  | BB-FS | FS-Ver | Vér_Har | Bud break | Fruit set | Veraison | Harvest |
| Means of main factors | Arg | 50.0±0.95 | 46.5±0.71 | 38.6±1.45 | 19-Mar±2.10 | 11-May±2.05 | 26-Jun±2.07 | 4-Aug±2.03 |
|  | Bar | 44.6±0.76 | 53.3±0.76 | 39.4±1.57 | 19-Mar±2.11 | 7-May±1.99 | 30-Jun±1.76 | 8-Aug±2.67 |
|  | CF | 48.6±1.18 | 54.4±0.71 | 37.1±1.25 | 17-Mar±1.96 | 9-May±2.16 | 2-Jul±1.80 | 9-Aug±2.03 |
|  | CS | 48.1±1.05 | 47.4±0.92 | 43.2±1.40 | 22-Mar±2.26 | 12-May±1.90 | 29-Jun±1.73 | 11-Aug±1.96 |
|  | Car | 49.0±1.14 | 51.3±1.03 | 47.9±1.29 | 17-Mar±2.21 | 9-May±2.23 | 29-Jun±2.08 | 16-Aug±2.17 |
|  | Dol | 46.4±0.85 | 44.7±0.93 | 41.0±1.60 | 20-Mar±2.06 | 9-May±1.97 | 23-Jun±2.26 | 3-Aug±2.34 |
|  | GN | 49.0±1.05 | 52.4±0.68 | 42.8±1.61 | 16-Mar±1.64 | 8-May±2.02 | 30-Jun±1.62 | 12-Aug±2.33 |
|  | Mal | 46.1±0.86 | 48.6±0.74 | 37.5±1.38 | 19-Mar±1.90 | 8-May±2.00 | 26-Jun±2.15 | 3-Aug±2.66 |
|  | Mer | 45.9±0.75 | 48.7±0.75 | 31.5±1.18 | 19-Mar±1.53 | 8-May±1.97 | 26-Jun±1.98 | 28-Jul±2.17 |
|  | PS | 47.3±0.94 | 46.2±0.61 | 41.1±1.29 | 20-Mar±1.95 | 10-May±1.89 | 25-Jun±1.73 | 5-Aug±2.28 |
|  | PV | 45.3±1.13 | 55.9±0.74 | 44.8±1.10 | 20-Mar±1.68 | 8-May±1.97 | 3-Jul±1.53 | 17-Aug±1.69 |
|  | PN | 42.8±0.63 | 46.6±0.56 | 36.3±1.47 | 17-Mar±2.27 | 3-May±2.21 | 19-Jun±2.00 | 25-Jul±2.47 |
|  | Pt | 49.9±1.23 | 47.6±1.04 | 45.0±0.78 | 20-Mar±1.96 | 12-May±2.13 | 29-Jun±1.92 | 13-Aug±1.96 |
|  | RC | 43.3±0.61 | 46.0±0.56 | 38.5±1.48 | 17-Mar±1.77 | 4-May±1.92 | 19-Jun±2.00 | 27-Jul±2.57 |
|  | Sg | 45.9±0.85 | 50.8±0.70 | 44.6±1.55 | 15-Mar±1.42 | 5-May±2.19 | 24-Jun±2.16 | 7-Aug±2.62 |
|  | Syrah | 48.0±0.93 | 47.7±0.70 | 38.9±1.68 | 21-Mar±1.94 | 11-May±1.85 | 28-Jun±1.90 | 6-Aug±2.45 |
|  | Tem | 46.4±1.34 | 42.6±0.87 | 45.8±2.95 | 23-Mar±2.27 | 12-May±2.29 | 23-Jun±1.89 | 9-Aug±2.03 |
|  | TC | 47.4±0.83 | 54.2±0.92 | 47.3±1.49 | 15-Mar±1.98 | 6-May±2.04 | 30-Jun±1.67 | 16-Aug±1.79 |
|  | TN | 44.1±1.32 | 49.0±0.65 | 44.1±2.24 | 23-Mar±2.00 | 10-May±2.25 | 28-Jun±2.02 | 11-Aug±2.62 |
|  | Zin | 44.8±1.07 | 48.9±0.64 | 44.9±0.91 | 21-Mar±1.93 | 9-May±2.02 | 26-Jun±2.02 | 11-Aug±1.53 |
|  | MR | 48.9±0.32 | 47.4±0.28 | 39.5±0.45 | 23-Mar±0.63 | 14-May±0.62 | 30-Jun±0.54 | 9-Aug±0.63 |
|  | RN | 44.4±0.26 | 50.9±0.33 | 43.5±0.61 | 15-Mar±0.49 | 3-May±0.51 | 23-Jun±0.66 | 6-Aug±0.95 |
|  | 2017 | 46.9±0.41 | 48.5±0.49 | 41.2±0.75 | 25-Mar±0.44 | 11-May±0.74 | 29-Jun±0.64 | 10-Aug±0.78 |
|  | 2018 | 46.4±0.26 | 49.3±0.35 | 40.9±0.65 | 13-Mar±0.32 | 28-Apr±0.41 | 16-Jun±0.46 | 27-Jul±0.78 |
|  | 2019 | - | 49.6±0.34 | 42.3±0.59 | - | 16-May±0.34 | 5-Jul±0.28 | 16-Aug±0.64 |
| ANOVA P of main factors and their interactions | Cultivar | <.0001 | <.0001 | <.0001 | <.0001 | <.0001 | <.0001 | <.0001 |
|  | Location | <.0001 | <.0001 | <.0001 | <.0001 | <.0001 | <.0001 | 0.0105 |
|  | Year | ns | <.0001 | 0.0050 | <.0001 | <.0001 | <.0001 | <.0001 |
|  | Location*year | <.0001 | <.0001 | <.0001 | <.0001 | <.0001 | <.0001 | 0.0042 |
|  | Cultivar*Location | 0.0152 | <.0001 | <.0001 | 0.0008 | <.0001 | <.0001 | <.0001 |
|  | Cultivar* Year | ns | <.0001 | <.0001 | <.0001 | <.0001 | <.0001 | <.0001 |
|  | Cultivar*Location*year | 0.0020 | <.0001 | <.0001 | ns | 0.0002 | <.0001 | 0.0047 |

Values are elapsed days mean ±SE, (*n* = 4 replicates x 2 location x 3 seasons). BB, Budbreak; FS, fruit set; Ver, véraison; Har, Harvest; RN, Ramat Negev; MR, Ramon. ns, not significant.

Table S5: Average difference of pulp organic acids and sugars (expressed in Δ mg g^-1^DW) between berry developmental stage in white and red cultivars Ramon (MR) and Ramat Negev (RN) from 2017-2019 seasons. Citrate is expressed in relative abundance.

| Cultivar | Years | Location | Difference between Vér and Har in organic acids | | | Difference between Har and Vér in sugars | | |
| --- | --- | --- | --- | --- | --- | --- | --- | --- |
|  |  |  | Malate | Tartrate | Citrate | Fructose | Glucose | Sucrose |
| Red | 2017 | RN | 124.8+7.1A* | 108.7+6.9A* | 58.2+6.1A | 68.9+4.2A | 20.0+3.2B | 45.3+4.1A |
|  | 2018 |  | 47.1+4.2B | 37.4+2.0B | 24.8+2.1B | 17.9+1.6C | 7.9+0.8C | 9.5+0.7B* |
|  | 2019 |  | 47.5+4.9B | 10.4+1.4C | 43.2+6.4A* | 44.1+7.3B | 46.3+8.0A* | 21.1+2.7B* |
|  |  | | | | | | | |
|  | 2017 | MR | 70.1+4.0a | 63.6+3.7a | 43.6+3.2a | 49.9+4.1a* | 15.2+2.7a | 41.1+4.4a |
|  | 2018 |  | 72.0+4.5a* | 53.3+2.5b* | 22.9+1.3b | 18.2+1.2b | 7.5+0.6a | 13.0+0.8b |
|  | 2019 |  | 39.0+3.6b | 10.7+1.3c | 16.0+1.7b | 31.5+8.1b | 22.2+6.6a | 13.4+3.7b |
|  | | | | | | | | |
| White | 2017 | RN | 61.2+10.5A* | 43.7+7.9A* | 27.4+5.4A* | 69.2+8.1A | 31.1+5.5B | 62.6+7.3A |
|  | 2018 |  | 14.5+1.9B | 21.1+2.4B | 15.8+1.2A | 12.0+1.2B | 6.2+0.5C | 10.0+1.3B |
|  | 2019 |  | 35.1+6.5AB | 8.1+2.6B | 28.9+5.8A | 46.9+10.1A | 58.3+12.2A* | 12.7+4.0B |
|  |  | | | | | | | |
|  | 2017 | MR | 7.4+0.8c | 10.4+1.7b | 8.9+2.1b | 66.1+7.5a | 27.8+3.7a | 60.9+5.9a |
|  | 2018 |  | 14.4+1.9b | 19.9+2.2a | 13.4+0.5b | 18.4+2.1b* | 8.1+1.2b | 15.2+1.2b* |
|  | 2019 |  | 45.1+5.0a | 10.6+1.9Ab | 22.1+3.5a | 29.1+8.1b | 20.7+6.5ab | 8.9+2.5b |

Data for 2017 and 2018 are the overall mean value ±SE of red (*n* = 4 replicates x 20 cultivars) and white (*n* = 4 replicates x 10 cultivars) cultivars at Ramon and Ramat Negev vineyards. Data of 2019 are the average value across all white (*n* = bulked replicate x 10 cultivars) and red (*n* = bulked replicate x 20 cultivars) cultivars at Ramon and Ramat Negev. *indicates a significant difference between locations within the same season. a, b, c; indicates significant differences between the seasons at Ramon vineyard. A, B, C; indicate significant differences between seasons at Ramat Negev vineyard. Ver, véraison; Har, Harvest.

Table S6: Malate, tartrate and citrate (expressed in mg g^-1^DW) accumulation at véraison in the pulp of white and red cultivar berries at Ramon (MR) and Ramat Negev (RN) from 2017-2019 growing seasons.

| Cultivar | | 2017 | | | | | | 2018 | | | | | | 2019 | | | | | |
| --- | --- | --- | --- | --- | --- | --- | --- | --- | --- | --- | --- | --- | --- | --- | --- | --- | --- | --- | --- |
|  |  | Malate | | Tartrate | | Citrate | | Malate | | Tartrate | | Citrate | | Malate | | Tartrate | | Citrate | |
| Red |  | MR | RN | MR | RN | MR | RN | MR | RN | MR | RN | MR | RN | MR | RN | MR | RN | MR | RN |
|  | Arg | 102.9 | **232.1** | 138.2 | 243.8 | 100.8 | 164.8 | 82.5 | 67.7 | 92.7 | 65.2 | 66.7 | 64.0 | 56.6 | 92.8 | 30.04 | 32.7 | 57.2 | 121.7 |
|  | Bar | 48.8 | **149.9** | 77.0 | 178.5 | 60.8 | **112.3** | 59.0 | 48.8 | 83.1 | 74.2 | 54.3 | 62.4 | 55.1 | 53.0 | 30.26 | 35.1 | 58.3 | 75.5 |
|  | CF | 60.0 | **95.1** | 79.7 | 97.1 | 70.6 | **76.1** | 65.7 | 53.0 | 59.6 | 53.4 | 51.1 | 60.9 | 24.6 | 37.9 | 17.37 | 17.1 | 39.2 | 44.9 |
|  | CS | 88.1 | 91.7 | 93.8 | 135.7 | 78.9 | 90.2 | **75.1** | 41.8 | 64.3 | 58.1 | 55.1 | 57.3 | 34.4 | 52.2 | 16.35 | 21.4 | 44.5 | 66.3 |
|  | Car | 112.6 | 139.5 | 116.4 | 136.2 | 92.0 | 82.7 | 92.3 | 78.2 | 91.5 | 73.1 | 50.5 | 52.4 | 66.2 | 57.6 | 33.93 | 30.3 | 33.5 | 65.2 |
|  | Dol | 65.8 | **128.2** | 74.3 | **132.5** | 68.2 | 86.1 | **43.8** | 14.7 | 53.2 | 35.1 | 48.7 | 49.2 | 72.5 | 60.5 | 28.82 | 23.1 | 59.6 | 88.5 |
|  | GN | 17.8 | **34.2** | 59.9 | 81.7 | 52.4 | 61.3 | **18.5** | 9.2 | **69.2** | 42.6 | 50.0 | 47.5 | 18.1 | 26.6 | 27.21 | 25.1 | 39.4 | 46.4 |
|  | Mal | 77.9 | **184.6** | 83.7 | 139.3 | 83.0 | 88.2 | 97.1 | 80.9 | **74.4** | 53.5 | 67.7 | 61.4 | 67.3 | 84.6 | 26.66 | 24.4 | 57.3 | 112.4 |
|  | Mer | 37.0 | 86.8 | 74.0 | **127.3** | 53.7 | 69.7 | 14.7 | 19.8 | 33.5 | **46.6** | 45.8 | 47.1 | 52.2 | 43.0 | 26.39 | 24.2 | 45.6 | 52.8 |
|  | PS | 62.2 | **189.0** | 109.0 | **207.5** | 68.3 | **125.5** | 84.1 | 58.1 | 99.1 | 82.7 | 59.8 | 77.6 | 24.4 | 71.1 | 16.98 | 35.3 | 42.6 | 124.7 |
|  | PV | 95.7 | 110.3 | 81.0 | 90.0 | 82.4 | **94.7** | **109.9** | 78.2 | 55.1 | 51.5 | 56.7 | 63.9 | 44.2 | 37.6 | 26.02 | 12.7 | 42.0 | 47.5 |
|  | PN | 76.0 | 88.1 | 78.0 | 73.8 | 64.0 | 65.8 | **96.8** | 31.3 | **61.1** | 35.8 | 57.5 | 50.4 | 48.5 | 46.1 | 20.08 | 16.8 | 50.4 | 59.6 |
|  | Pt | 143.0 | **216.9** | 98.4 | **159.9** | 100.8 | 127.2 | 166.3 | 154.6 | 76.0 | 64.8 | 67.5 | 77.2 | - | 95.8 | - | 24.1 | - | 131.5 |
|  | RC | 118.2 | 86.8 | 97.2 | 90.9 | **92.9** | 67.4 | 71.1 | 54.6 | **66.9** | 44.3 | 54.5 | 58.0 | 28.8 | 41.0 | 31.89 | 19.5 | 57.9 | 58.9 |
|  | Sg | 32.0 | **82.7** | 96.0 | 148.7 | 62.4 | 70.4 | **40.3** | 18.0 | 89.5 | 66.9 | 52.1 | 48.6 | 49.3 | 31.4 | 17.80 | 29.9 | 44.1 | 62.1 |
|  | Syrah | 95.8 | 103.3 | 96.7 | 143.8 | 78.7 | 93.1 | 112.8 | 73.8 | 62.7 | 51.5 | 60.9 | 63.2 | 39.1 | 95.4 | 15.83 | 26.4 | 49.3 | 94.9 |
|  | Temp | 101.1 | 129.9 | 70.7 | 100.4 | 85.1 | 77.3 | **84.0** | 42.5 | 40.6 | 31.2 | 55.5 | 53.7 | 42.9 | 69.4 | 20.73 | 20.6 | 43.1 | 65.0 |
|  | TC | 99.3 | **146.1** | 82.1 | **117.4** | 82.8 | 109.0 | 113.4 | 115.9 | 73.9 | 54.5 | 56.1 | 78.5 | 61.5 | 61.1 | 17.21 | 22.9 | 46.9 | 106.9 |
|  | TN | 85.1 | **289.0** | 55.0 | **116.2** | 72.4 | 130.1 | **119.9** | 58.2 | 48.0 | 42.6 | 69.8 | 58.5 | 38.6 | 45.1 | 19.07 | 15.1 | 51.2 | 61.7 |
|  | Zin | 68.4 | 139.0 | 67.3 | 143.7 | 68.7 | 82.6 | **68.3** | 26.0 | **66.3** | 46.0 | 56.4 | 52.4 | 56.6 | 31.8 | 30.04 | 23.0 | 43.4 | 56.0 |
|  | Average | 79.4 | **134.1** | 86.4 | **133.6** | 75.9 | **93.7** | **80.8** | 56.2 | **68.0** | 53.8 | 56.8 | 59.2 | 46.5 | 56.7 | 23.5 | 24.0 | 47.6 | **77.1** |
|  |  |  |  |  |  |  |  |  |  |  |  |  |  |  |  |  |  |  |  |
| White  White | Chardonnay | 19.3 | **90.8** | 29.3 | **63.0** | 41.2 | 54.6 | 23.9 | 17.5 | 31.5 | 27.5 | 48.0 | 45.4 | 53.8 | 58.7 | 15.1 | 20.3 | 44.9 | 62.9 |
|  | Chenin B | 14.7 | **54.6** | 27.9 | **58.9** | 51.7 | 68.1 | 21.2 | 20.5 | 37.3 | 38.1 | 46.9 | 54.1 | 19.0 | 39.4 | 13.7 | 19.3 | 47.9 | 26.3 |
|  | Colombard | 22.4 | 183.5 | 59.0 | **164.2** | 50.2 | 94.8 | 26.7 | 35.1 | 49.1 | 58.9 | 44.7 | 51.9 | 69.2 | 74.8 | 41.4 | 36.3 | 60.2 | 64.3 |
|  | Gewurzt | 13.6 | 79.3 | 33.9 | 47.8 | 42.2 | 63.3 | 10.2 | 10.3 | **16.7** | 11.5 | 43.6 | 40.4 | 34.6 | 55.7 | 11.3 | 21.5 | 53.5 | 43.0 |
|  | Muscat A | 15.4 | 15.0 | 34.1 | 28.4 | 47.1 | 47.7 | 43.8 | 17.9 | 51.0 | 52.9 | 45.7 | 50.0 | 33.0 | 41.8 | 24.5 | 27.9 | 45.5 | 60.4 |
|  | Muscat B | 9.7 | 103.8 | 25.8 | 103.5 | 43.2 | **90.4** | 13.3 | 7.1 | 25.5 | 31.9 | 44.0 | 42.1 | 38.5 | 59.6 | 22.0 | 29.4 | 52.7 | 49.4 |
|  | Pinot G | 12.9 | **112.2** | 27.2 | **54.6** | 41.2 | **69.2** | 32.7 | 41.1 | 31.7 | 30.1 | 49.8 | 48.3 | 27.6 | 49.9 | 15.2 | 20.0 | 47.4 | 32.0 |
|  | Semillon | 11.9 | 17.9 | 27.2 | 25.0 | 36.7 | 42.9 | 21.5 | 24.7 | 35.3 | 41.1 | 46.2 | 53.5 | 38.4 | 30.3 | 16.2 | 17.2 | 44.3 | 61.2 |
|  | Sauvignon B | 15.5 | **64.0** | 28.5 | 87.8 | 40.3 | **64.4** | 19.5 | **32.7** | 32.6 | 25.2 | 48.3 | 54.0 | 50.6 | 79.9 | 18.9 | 32.0 | 80.3 | 74.7 |
|  | Riesling | 20.5 | 20.3 | **49.2** | 31.1 | 49.5 | 43.7 | 24.4 | 36.3 | 55.2 | 57.0 | 46.8 | 51.4 | 83.4 | 38.2 | 27.0 | 19.8 | 43.1 | 93.8 |
|  | Average | 15.6 | **74.2** | 34.2 | **66.4** | 44.4 | **63.9** | 23.7 | 24.3 | 36.6 | 37.4 | 46.4 | 49.1 | 52.8 | 44.8 | 24.4 | 20.5 | 52.0 | 56.8 |

Cultivar means in bold represent significant differences between locations within the cultivar based on t test. See Supp.1. Data for 2017 and 2018 are means of four biological replicates; results were validated in 2019 using bulked replicate.

Table S7: Malate, tartrate and citrate (expressed in mg g^-1^DW) accumulation at harvest in the pulp of white and red cultivar berries at Ramon (MR) and Ramat Negev (RN) from 2017-2019 seasons.

| cultivar | | 2017 | | | | | | 2018 | | | | | | 2019 | | | | | |
| --- | --- | --- | --- | --- | --- | --- | --- | --- | --- | --- | --- | --- | --- | --- | --- | --- | --- | --- | --- |
|  |  | Malate | | Tartrate | | Citrate | | Malate | | Tartrate | | Citrate | | Malate | | Tartrate | | Citrate | |
| Red |  | MR | RN | MR | RN | MR | RN | MR | RN | MR | RN | MR | RN | MR | RN | MR | RN | MR | RN |
|  | Arg | 10.8 | 10.4 | 14.6 | 19.3 | 27.9 | 32.4 | 8.8 | 11.1 | 14.3 | 13.2 | 34.5 | 34.0 | 7.6 | 10.7 | 14.3 | 12.4 | 29.8 | 33.2 |
|  | Bar | 10.2 | 7.7 | 38.4 | 30.1 | 36.0 | 30.9 | 8.2 | 7.1 | 21.8 | 27.0 | 33.8 | 34.0 | 6.8 | 7.6 | 15.7 | 14.2 | 33.0 | 33.3 |
|  | CF | 7.2 | 7.5 | 17.4 | 22.3 | 25.1 | 34.2 | 7.1 | 6.7 | 15.4 | 15.7 | 31.9 | 31.6 | 5.8 | 8.0 | 12.7 | 12.4 | 28.2 | 31.7 |
|  | CS | 8.0 | 7.4 | 22.6 | 20.8 | 36.4 | 36.4 | 7.7 | **9.5** | 16.5 | 15.0 | 35.4 | 35.8 | 8.7 | 7.4 | 11.9 | 13.9 | 35.8 | 34.9 |
|  | Car | 10.5 | 8.6 | 35.2 | 24.4 | 43.5 | 35.0 | 9.2 | 10.5 | 17.6 | 20.1 | 34.4 | 34.8 | 7.1 | 9.7 | 13.0 | 12.6 | 31.0 | 34.6 |
|  | Dol | 4.8 | 8.3 | 15.1 | 30.0 | 19.4 | 33.4 | 6.9 | 5.8 | 18.8 | 17.3 | 30.8 | 31.6 | 5.4 | 5.7 | 11.2 | 14.1 | 29.6 | 30.9 |
|  | GN | 5.9 | 6.4 | 23.5 | 33.4 | 25.5 | 29.4 | 4.7 | 6.2 | 19.3 | 22.6 | 31.0 | 31.6 | 5.1 | 5.6 | 15.1 | 16.0 | 28.0 | 30.5 |
|  | Mal | 8.8 | 7.5 | 26.7 | 16.9 | 39.6 | 29.1 | 11.0 | 11.5 | 9.1 | 13.1 | 35.6 | 35.6 | 8.6 | 13.7 | 11.8 | 18.9 | 32.7 | 33.2 |
|  | Mer | 6.5 | 5.1 | 27.9 | 18.4 | 30.0 | 22.0 | 6.3 | 7.1 | **20.0** | 14.2 | 31.0 | 31.9 | 6.3 | 6.3 | 13.1 | 13.2 | 28.2 | 30.9 |
|  | PS | 10.2 | 10.3 | 20.8 | 27.7 | 36.1 | 49.1 | 10.1 | 8.6 | 13.1 | 16.2 | 34.0 | 35.5 | 9.5 | 6.0 | 13.6 | 13.0 | 32.1 | 33.8 |
|  | PV | 6.1 | 12.6 | 14.7 | 23.6 | 24.8 | 44.9 | 8.2 | 9.0 | 15.1 | 14.9 | 33.6 | 33.8 | 6.2 | 11.5 | 13.0 | 11.1 | 30.7 | 36.0 |
|  | PN | 8.2 | 15.9 | 18.0 | 26.9 | 29.7 | 47.7 | 8.9 | 11.7 | 11.0 | 12.4 | 35.4 | **37.7** | 7.8 | 5.7 | 11.5 | 11.7 | 34.1 | 31.1 |
|  | Pt | 11.8 | 8.9 | 28.6 | 30.6 | 33.6 | 31.7 | 9.1 | 11.0 | 15.8 | 17.4 | 33.8 | 36.3 | 9.8 | 6.9 | 15.3 | 14.1 | 30.5 | 34.3 |
|  | RC | 7.5 | 11.5 | 14.8 | 18.5 | 29.2 | 35.5 | 11.3 | 12.4 | 11.5 | **17.1** | 36.8 | 36.0 | 8.8 | 15.7 | 11.2 | 12.7 | 35.0 | 37.7 |
|  | Sg | 7.0 | 5.5 | 19.4 | 17.5 | 33.9 | 25.4 | 6.6 | 6.5 | 12.5 | **18.3** | 33.9 | 32.8 | 6.2 | 8.0 | 11.9 | 12.0 | 31.2 | 34.8 |
|  | Syrah | 12.4 | 8.1 | 21.6 | 26.6 | 38.8 | 38.8 | **13.2** | 10.2 | 10.9 | 13.9 | 35.3 | 35.5 | 8.6 | 9.4 | 12.6 | 14.9 | 33.7 | 34.7 |
|  | Temp | 11.7 | 14.1 | 27.5 | 31.2 | 31.9 | 40.7 | 9.8 | 8.9 | 12.7 | 16.5 | 33.4 | 33.8 | 9.9 | 13.6 | 14.2 | 14.1 | 34.0 | 38.0 |
|  | TC | 9.4 | 8.8 | 23.6 | 22.1 | 34.4 | 32.3 | 7.5 | 8.2 | 11.5 | 15.5 | 35.0 | 32.5 | 7.6 | 7.3 | 11.8 | 14.0 | 31.6 | 33.5 |
|  | TN | 17.1 | 11.5 | 19.2 | 18.9 | 38.4 | 35.9 | 11.5 | 11.8 | 11.2 | 10.2 | 35.2 | 41.3 | 9.5 | 17.5 | 11.5 | 12.7 | 33.0 | 37.8 |
|  | Zin | 12.6 | 10.2 | 27.3 | 36.0 | 32.7 | 45.0 | 9.6 | 9.9 | 15.5 | 15.9 | **34.0** | 32.2 | 6.3 | 8.1 | 13.5 | 13.3 | 29.9 | 33.3 |
|  | Average | 9.3 | 9.3 | 22.8 | 24.8 | 32.4 | 35.5 | 8.8 | 9.2 | 14.7 | **16.4** | 33.9 | 34.4 | 7.6 | 9.2 | 12.9 | 13.6 | 31.6 | **33.9** |
| White |  |  |  |  |  |  |  |  |  |  |  |  |  |  |  |  |  |  |  |
|  | Chardonnay | 10.8 | 19.0 | 21.7 | 31.6 | 37.1 | 50.1 | 14.4 | 12.2 | **19.9** | 12.8 | 35.0 | 31.7 | 9.2 | 12.6 | 11.1 | 10.7 | 30.2 | 33.2 |
|  | Chenin B | 9.2 | 9.3 | 25.6 | 22.1 | 45.8 | 35.0 | 7.8 | 9.5 | 19.8 | 14.6 | 34.1 | 33.9 | 7.4 | 8.4 | 13.7 | 10.6 | 28.5 | 32.5 |
|  | Colombard | 12.2 | 22.9 | 32.9 | 21.4 | 33.9 | **48.1** | 13.9 | **19.0** | 15.7 | **22.0** | 33.4 | 34.4 | 9.0 | 10.4 | 20.4 | 14.2 | 30.1 | 32.2 |
|  | Gewurzt | 6.9 | 11.6 | 18.0 | 18.9 | 27.0 | 32.7 | 5.1 | 6.7 | **16.0** | 7.8 | 30.9 | 30.9 | 6.3 | 6.0 | 11.6 | 10.8 | 29.5 | 29.5 |
|  | Muscat A | **7.8** | 4.8 | **16.5** | 11.0 | **26.3** | 20.3 | **11.5** | 7.0 | **15.0** | 9.5 | **32.5** | 31.4 | 7.6 | 15.7 | 13.3 | 14.7 | 29.4 | 33.4 |
|  | Muscat B | 6.0 | 11.7 | 22.2 | 25.1 | 39.1 | 40.0 | **6.8** | 4.5 | 16.2 | **24.0** | 31.6 | 32.2 | 8.6 | 6.7 | 14.1 | 12.3 | 30.3 | 31.8 |
|  | Pinot G | 8.1 | 12.3 | 26.0 | 23.9 | 38.3 | 38.4 | 8.7 | 10.4 | 11.4 | 13.2 | 33.3 | 34.5 | 6.8 | 12.5 | 13.9 | 13.4 | 31.4 | 29.9 |
|  | Semillon | 5.1 | 12.1 | 18.6 | 19.2 | 27.2 | 40.2 | 8.2 | 10.0 | 20.5 | 16.8 | 31.5 | 33.5 | 6.9 | 11.6 | 13.5 | 13.7 | 29.5 | 28.9 |
|  | Sauvignon B | 6.2 | 10.2 | 23.5 | 26.8 | 39.6 | 43.7 | 9.5 | 9.1 | 10.9 | 11.3 | 36.4 | 36.1 | 7.3 | 7.7 | 10.8 | 12.4 | 30.4 | 30.7 |
|  | Riesling | 10.2 | 15.4 | 32.7 | 27.3 | 40.6 | 50.3 | 7.2 | 9.9 | 22.0 | 31.2 | 31.8 | **34.8** | 8.1 | 6.9 | 15.4 | 12.0 | 29.4 | 31.8 |
|  | Average | 8.2 | **12.9** | 23.8 | 22.8 | 35.5 | 39.9 | 9.3 | 9.8 | 16.7 | 16.3 | 33.1 | 33.1 | 7.7 | 9.8 | 13.8 | 12.5 | 29.9 | **31.4** |

Cultivar means in bold represent significant differences between locations within the cultivar based on t test. See Supp.1. Data for 2017 and 2018 are means of four biological replicates; results were validated in 2019 using bulked replicate

Table S8: Sugar accumulations (expressed in mg g^-1^DW) at véraison in the pulp of red and white cultivar berries at Ramon (MR) and Ramat Negev (RN) from 2017-2019 seasons.

| cultivar | | 2017 | | | | | | 2018 | | | | | | 2019 | | | | | |
| --- | --- | --- | --- | --- | --- | --- | --- | --- | --- | --- | --- | --- | --- | --- | --- | --- | --- | --- | --- |
|  |  | Fructose | | Glucose | | Sucrose | | Fructose | | Glucose | | Sucrose | | Fructose | | Glucose | | Sucrose | |
| Red |  | MR | RN | MR | RN | MR | RN | MR | RN | MR | RN | MR | RN | MR | RN | MR | RN | MR | RN |
|  | Arg | 31.4 | 27.6 | 39.7 | 48.9 | 37.4 | 27.7 | 39.8 | 45.1 | 45.0 | 47.2 | 51.5 | 46.9 | 47.5 | 46.3 | 34.1 | 52.5 | 49.1 | 45.2 |
|  | Bar | 41.9 | 37.2 | 42.1 | 47.5 | **47.7** | 40.3 | 43.3 | 49.3 | 43.6 | 47.4 | 46.7 | 47.8 | 49.4 | 56.4 | 28.4 | 59.2 | 53.2 | 46.4 |
|  | CF | 44.0 | 42.2 | 46.1 | 50.4 | **40.0** | 34.1 | 40.8 | 44.2 | 45.3 | 45.8 | 43.0 | 44.0 | 59.9 | 52.9 | 40.1 | 47.0 | 46.5 | 44.4 |
|  | CS | 48.5 | 39.5 | 49.1 | 50.8 | **45.3** | 36.3 | 38.9 | 46.3 | 43.4 | 47.8 | 46.3 | 46.8 | 46.2 | 61.0 | 31.2 | 35.3 | 51.4 | 59.0 |
|  | Car | 38.0 | 40.0 | 43.3 | 50.9 | 46.4 | 45.3 | 47.7 | 46.8 | 48.9 | 48.6 | 54.5 | 48.2 | 53.8 | 49.1 | 30.2 | 48.7 | 69.9 | 48.5 |
|  | Dol | 43.1 | 40.1 | 43.7 | 50.8 | 38.8 | 36.9 | 38.6 | 44.7 | 41.6 | 45.2 | 43.5 | 40.4 | 54.2 | 43.3 | 26.6 | 48.4 | 44.3 | 36.8 |
|  | GN | 53.7 | 46.6 | 45.1 | 50.9 | 42.7 | 43.3 | 40.7 | **50.0** | 43.1 | 46.8 | 45.3 | 49.7 | 51.6 | 51.5 | 33.0 | 47.6 | 41.3 | 45.2 |
|  | Mal | 39.8 | 38.5 | 43.7 | 48.5 | 46.5 | 43.6 | 37.8 | 43.6 | 45.1 | 46.6 | 50.5 | 50.3 | 58.4 | 45.5 | 29.8 | 47.8 | 55.0 | 45.8 |
|  | Mer | 48.5 | 45.4 | 47.8 | 52.6 | 47.7 | 44.6 | 46.6 | 46.5 | 45.7 | 46.7 | 48.8 | 47.3 | 48.8 | 47.6 | 34.4 | 42.0 | 47.7 | 45.9 |
|  | PS | 35.6 | 34.8 | 41.1 | 49.7 | 40.3 | 37.3 | 39.8 | 45.8 | 45.6 | 47.5 | 43.5 | 47.7 | 12.1 | 49.9 | 5.9 | 59.4 | 23.4 | 44.9 |
|  | PV | 39.1 | 40.3 | 43.4 | 49.0 | 41.8 | 37.8 | 39.0 | 48.3 | 44.0 | 49.4 | 42.8 | 43.2 | 81.0 | 50.7 | 56.6 | 42.6 | 46.5 | 44.2 |
|  | PN | 41.3 | 53.4 | 43.4 | **53.6** | 41.0 | **54.2** | 49.0 | 47.2 | 47.9 | 47.0 | 45.0 | 49.1 | 52.9 | 53.9 | 30.2 | 51.3 | 50.1 | 45.4 |
|  | Pt | 35.3 | 32.1 | 42.6 | 49.5 | **39.7** | 28.0 | 38.6 | 47.5 | 44.9 | 47.4 | 40.7 | 45.8 | - | 39.9 | - | 42.1 | - | 40.6 |
|  | RC | 37.0 | **45.4** | 42.6 | 49.9 | 42.0 | **52.9** | 45.4 | 41.2 | 45.7 | 45.5 | 48.3 | 51.1 | 47.7 | 47.4 | 32.6 | 45.0 | 46.7 | 45.0 |
|  | Sg | 42.3 | 44.1 | 43.3 | 51.2 | **44.3** | 42.8 | 43.3 | 49.2 | 45.0 | 47.2 | 49.0 | 49.2 | 50.9 | 42.7 | 34.9 | 37.5 | 48.5 | 41.9 |
|  | Syrah | 41.9 | 37.0 | 44.7 | 50.7 | **39.7** | 32.9 | 35.3 | 42.7 | 43.7 | 44.7 | 47.7 | 47.6 | 50.6 | 60.9 | 32.7 | 70.5 | 54.7 | 51.1 |
|  | Temp | 37.7 | 41.0 | 42.7 | 49.6 | 42.7 | 42.9 | 40.0 | 47.5 | 43.5 | 47.5 | **48.6** | 42.7 | 50.9 | 48.5 | 34.5 | 49.9 | 55.1 | 48.8 |
|  | TC | 36.4 | 36.1 | 44.1 | 50.8 | 34.3 | 33.1 | 42.7 | 36.8 | 48.0 | 44.1 | **43.3** | 35.2 | 44.0 | 41.8 | 29.0 | 42.5 | 36.1 | 35.2 |
|  | TN | **47.9** | 29.1 | 45.9 | 46.8 | **50.7** | 39.3 | 39.2 | 42.8 | 43.6 | 46.4 | 52.5 | 48.6 | 54.0 | 46.0 | 37.4 | 39.9 | 57.0 | 48.9 |
|  | Zin | 37.4 | 41.8 | 42.0 | 52.0 | 37.9 | 43.1 | 38.7 | 46.0 | 43.2 | 46.3 | 49.4 | 46.3 | 43.5 | 49.7 | 28.8 | 45.3 | 48.0 | 42.5 |
|  | Average | 41.0 | 39.8 | 43.8 | **50.2** | **42.4** | 39.9 | 41.3 | **45.6** | 44.8 | **46.8** | 47.0 | 46.4 | 50.4 | 49.3 | 32.1 | **47.7** | **48.7** | 45.3 |
|  |  |  |  |  |  |  |  |  |  |  |  |  |  |  |  |  |  |  |  |
| White | Chardonnay | 59.3 | 41.5 | 48.6 | 36.3 | 50.5 | 36.3 | 46.4 | 49.2 | 45.5 | 46.2 | 50.4 | 50.0 | 53.1 | 44.6 | 32.8 | 34.0 | 48.7 | 50.7 |
|  | Chenin B | 51.6 | 57.2 | 46.4 | **64.0** | 56.3 | 59.2 | 50.4 | 47.0 | **48.6** | 45.9 | **51.6** | 45.7 | 50.0 | 43.4 | 33.9 | 33.0 | 49.6 | 48.6 |
|  | Colombard | **60.5** | 41.3 | 50.1 | 53.7 | **63.4** | 46.7 | 45.1 | 46.5 | 45.2 | 45.9 | 54.2 | 49.8 | 47.9 | 58.1 | 30.3 | 68.3 | 50.1 | 57.2 |
|  | Gewurzt | 49.1 | 44.3 | 46.7 | 50.4 | 39.2 | 51.6 | 46.3 | 48.0 | 45.6 | 44.7 | 56.5 | 48.7 | 47.5 | 38.1 | 32.0 | 36.1 | 52.9 | 48.0 |
|  | Muscat A | 57.3 | 50.9 | 47.4 | 49.3 | 49.9 | 43.1 | 45.8 | 49.3 | 47.9 | 47.5 | 46.1 | 51.7 | 49.3 | 41.4 | 35.1 | 35.2 | 49.4 | 46.5 |
|  | Muscat B | 56.7 | 47.5 | 44.9 | 54.6 | 51.9 | 42.3 | 44.9 | 48.6 | 42.2 | 45.5 | 47.7 | 49.8 | 52.6 | 42.4 | 34.3 | 31.8 | 55.0 | 48.7 |
|  | Pinot G | 58.9 | 49.0 | 46.8 | 49.8 | 50.0 | 42.7 | 43.6 | 46.2 | 44.9 | 46.3 | 44.1 | **50.6** | 51.4 | 65.0 | 31.0 | 60.8 | 50.0 | 62.3 |
|  | Semillon | 49.7 | 51.1 | 42.3 | 51.0 | **58.9** | 49.0 | 43.0 | 48.8 | 43.9 | 47.3 | 53.2 | 47.0 | 52.5 | 41.3 | 28.5 | 32.2 | 53.3 | 47.3 |
|  | Sauvignon B | 56.3 | 49.1 | 44.2 | 52.2 | 50.8 | 51.6 | 45.9 | 46.5 | 45.3 | 45.8 | 57.4 | 53.2 | 43.8 | 42.7 | 32.5 | 42.2 | 44.0 | 53.9 |
|  | Riesling | 49.1 | 45.8 | 45.0 | 46.8 | 49.0 | 47.9 | 41.2 | 46.9 | 44.8 | 45.7 | 48.0 | 47.0 | 47.3 | 47.5 | 26.7 | 47.7 | 50.1 | 46.5 |
|  | Average | **54.9** | 47.8 | 46.2 | **50.8** | **51.9** | 47.0 | 45.3 | 47.7 | 45.4 | 46.1 | 50.9 | 49.3 | 49.5 | 46.4 | 31.7 | **42.1** | 50.3 | 51.0 |

Cultivar means in bold represent significant differences between locations within the cultivar based on t test. See Supp.1. Data for 2017 and 2018 are means of four biological replicates; results were validated in 2019 using bulked replicate.

Table S9: Sugar accumulations (expressed in mg g^-1^DW) at harvest in the pulp of red and white cultivar berries at Ramon (MR) and Ramat Negev (RN) from 2017-2019 seasons.

| cultivar | | 2017 | | | | | | 2018 | | | | | | 2019 | | | | | |
| --- | --- | --- | --- | --- | --- | --- | --- | --- | --- | --- | --- | --- | --- | --- | --- | --- | --- | --- | --- |
|  |  | Fructose | | Glucose | | Sucrose | | Fructose | | Glucose | | Sucrose | | Fructose | | Glucose | | Sucrose | |
| Red |  | MR | RN | MR | RN | MR | RN | MR | RN | MR | RN | MR | RN | MR | RN | MR | RN | MR | RN |
|  | Arg | 67.3 | 97.1 | 46.4 | 62.3 | 73.7 | 92.1 | 57.2 | 55.5 | 51.4 | 51.7 | 61.8 | 55.0 | 85.8 | 76.0 | 60.8 | 82.7 | 68.1 | 59.5 |
|  | Bar | 106.5 | 89.1 | 64.5 | 53.6 | 106.8 | 62.0 | 56.7 | 64.4 | 53.8 | 54.7 | **59.7** | 51.6 | 60.2 | 110.9 | 35.8 | 115.3 | 54.6 | 68.2 |
|  | CF | 73.9 | 102.8 | 47.3 | 60.8 | 59.9 | 68.1 | 72.7 | 64.0 | 57.2 | 54.4 | 54.4 | 48.1 | 76.0 | 59.5 | 48.8 | 52.7 | 59.0 | 47.6 |
|  | CS | 91.6 | 100.6 | 56.8 | 57.9 | 82.1 | 76.4 | 58.9 | 57.1 | 51.8 | 51.9 | **66.3** | 56.7 | 59.3 | 118.5 | 34.8 | 132.5 | 63.3 | 74.1 |
|  | Car | 112.1 | 112.5 | 79.2 | 74.2 | 131.5 | 112.2 | 57.7 | 61.5 | 52.0 | 53.6 | 65.2 | 64.0 | 58.8 | 55.2 | 33.6 | 49.8 | 69.9 | 58.8 |
|  | Dol | 49.3 | **112.8** | 37.3 | **69.9** | 44.0 | 76.6 | 60.2 | 65.5 | 51.4 | 55.3 | 53.7 | 47.3 | 55.4 | 75.8 | 29.0 | 71.1 | 46.8 | 46.6 |
|  | GN | 91.3 | 110.1 | 54.4 | 68.6 | 72.3 | 71.2 | 63.6 | 69.9 | 55.6 | 58.2 | 48.7 | 55.6 | 92.3 | 92.9 | 63.1 | 91.3 | 41.8 | 55.9 |
|  | Mal | 88.6 | 88.9 | 60.4 | 60.2 | 88.3 | 90.2 | 59.6 | 66.3 | 52.2 | 54.1 | 65.9 | 67.1 | 58.5 | 166.9 | 31.9 | 197.3 | 61.8 | 94.1 |
|  | Mer | **144.6** | 76.3 | 76.9 | 53.2 | 89.5 | 66.4 | 65.8 | 64.7 | 55.3 | 55.1 | 64.2 | 56.5 | 118.8 | 84.5 | 94.1 | 91.4 | 51.0 | 65.3 |
|  | PS | 84.7 | 123.2 | 60.4 | 92.6 | 86.3 | 115.2 | 58.5 | 59.4 | 53.5 | 53.3 | 67.5 | 57.9 | 105.1 | 100.7 | 77.0 | 101.9 | 67.2 | 82.4 |
|  | PV | 62.8 | **117.4** | 45.3 | 87.5 | 44.3 | **88.4** | 68.3 | 70.3 | 55.5 | 59.9 | **57.2** | 46.8 | 88.2 | 55.3 | 57.1 | 52.7 | 47.7 | 51.8 |
|  | PN | 91.0 | **149.5** | 60.1 | 100.2 | 79.9 | 114.5 | 57.1 | 60.4 | 51.3 | 53.7 | 58.4 | 59.4 | 61.9 | 111.3 | 30.9 | 114.8 | 60.3 | 73.2 |
|  | Pt | 102.2 | 95.2 | 62.5 | 61.6 | 91.3 | 85.0 | 54.9 | 60.8 | 48.7 | 53.8 | 60.3 | 60.4 | 147.1 | 109.7 | 115.8 | 118.8 | 68.0 | 86.2 |
|  | RC | 71.3 | 96.9 | 43.5 | 58.5 | 65.0 | 86.9 | 55.9 | 57.4 | 50.0 | 51.3 | 66.9 | 61.6 | 59.8 | 77.7 | 34.4 | 78.3 | 67.4 | 74.7 |
|  | Sg | 103.0 | 90.9 | 63.2 | 55.2 | 99.6 | 65.6 | 56.9 | 65.5 | 48.9 | 55.4 | 62.2 | 57.1 | 64.5 | 61.5 | 37.2 | 53.0 | 63.3 | 54.8 |
|  | Syrah | 104.9 | 114.4 | 69.2 | 73.7 | 96.9 | 94.7 | 57.8 | 58.0 | 51.6 | 52.3 | 60.3 | 58.3 | 85.2 | 183.4 | 56.2 | 147.0 | 65.2 | 71.8 |
|  | Temp | 82.7 | 134.9 | 53.7 | 82.4 | 87.4 | 91.2 | 53.8 | 62.8 | 51.4 | 54.1 | 55.2 | 48.4 | 98.8 | 68.6 | 66.6 | 62.4 | 66.2 | 64.2 |
|  | TC | 107.7 | 113.2 | 76.1 | 69.5 | 95.5 | 64.7 | 56.5 | 60.2 | 50.3 | 52.4 | **54.3** | 42.4 | 58.0 | 106.5 | 32.4 | 110.7 | 49.6 | 53.1 |
|  | TN | 83.1 | 98.0 | 61.2 | 61.6 | 82.5 | 75.7 | 57.6 | 84.5 | 51.5 | 63.0 | 55.4 | 60.8 | 85.2 | 76.8 | 56.2 | 77.9 | 58.2 | 78.2 |
|  | Zin | 99.5 | 138.0 | 62.0 | 86.8 | 92.3 | 95.5 | 59.7 | 66.3 | 52.9 | 56.7 | 62.8 | 60.9 | 67.9 | 74.8 | 58.0 | 78.2 | 62.8 | 67.8 |
|  | Average | 90.9 | **108.2** | 59.0 | **69.6** | 83.4 | 84.7 | 59.5 | 63.5 | 52.3 | 54.6 | **60.0** | 55.7 | 79.3 | 93.3 | 52.7 | **94.0** | 59.6 | 66.4 |
|  |  |  |  |  |  |  |  |  |  |  |  |  |  |  |  |  |  |  |  |
| White | Chardonnay | 141.7 | 137.0 | 69.3 | 98.2 | 105.4 | 141.4 | 62.7 | 56.8 | 52.7 | 51.4 | **69.0** | 58.4 | 54.1 | 61.0 | 33.1 | 64.1 | 58.7 | 63.0 |
|  | Chenin B | 134.9 | 95.0 | 90.6 | 74.9 | 126.5 | 84.2 | 67.9 | 58.4 | 56.5 | 51.6 | **77.4** | 57.2 | 100.1 | 55.8 | 70.6 | 54.9 | 57.7 | 61.6 |
|  | Colombard | 108.1 | 153.2 | 69.7 | 93.4 | 113.9 | 160.4 | 59.5 | 61.2 | 52.2 | 53.9 | 71.4 | 71.3 | 69.4 | 91.1 | 66.0 | 98.8 | 52.1 | 91.3 |
|  | Gewurzt | 100.8 | 101.2 | 59.4 | 72.9 | 96.6 | 112.7 | 68.9 | 58.7 | 56.1 | 52.3 | 70.4 | 59.5 | 60.6 | 104.7 | 35.9 | 113.0 | 66.7 | 73.3 |
|  | Muscat A | 93.5 | 69.2 | 55.4 | 44.7 | 100.3 | 70.4 | 60.4 | 65.4 | 51.6 | 54.4 | 65.7 | 60.6 | 65.3 | 58.6 | 35.7 | 58.0 | 68.1 | 53.3 |
|  | Muscat B | 143.7 | 118.3 | 77.0 | 73.8 | 115.4 | 101.8 | 68.5 | 59.3 | 56.1 | 51.6 | 55.2 | 56.3 | 82.3 | 73.0 | 53.0 | 68.0 | 63.3 | 57.3 |
|  | Pinot G | 161.4 | 114.5 | 87.7 | 73.3 | 124.0 | 88.5 | 59.8 | 63.2 | 50.4 | 53.4 | 55.6 | 53.5 | 71.4 | 151.5 | 38.3 | 177.3 | 51.4 | 48.1 |
|  | Semillon | 92.5 | 119.1 | 66.8 | 82.7 | 94.3 | 90.0 | 76.8 | 57.9 | 59.2 | 51.7 | **65.0** | 58.4 | 53.3 | 149.1 | 33.0 | 160.9 | 55.6 | 57.6 |
|  | Sauvignon B | 119.8 | 111.7 | 85.9 | 87.1 | 140.6 | 127.0 | 54.4 | 53.0 | 50.0 | 49.3 | 68.8 | 66.4 | 113.4 | 91.8 | 74.7 | 104.2 | 68.5 | 71.2 |
|  | Riesling | 113.6 | 150.4 | 78.9 | 118.0 | 100.6 | 119.9 | 57.6 | 63.5 | 49.8 | 53.4 | **62.8** | 51.4 | 116.6 | 96.8 | 83.5 | 104.7 | 50.4 | 59.7 |
|  | Average | 121.0 | 117.0 | 74.1 | 81.9 | 111.8 | 109.7 | 63.6 | 59.7 | 53.4 | 52.3 | **66.1** | 59.3 | 78.7 | 93.3 | 52.4 | **100.4** | 59.2 | 63.6 |

Cultivar means in bold represent significant differences between locations within the cultivar based on t test. See Supp.1. Data for 2017 and 2018 are means of four biological replicates; results were validated in 2019 using bulked replicate.
